# Predicting health insurance uptake in Kenya using Random Forest: An analysis of socio-economic and demographic factors

**DOI:** 10.1371/journal.pone.0294166

**Published:** 2023-11-30

**Authors:** Nelson Kimeli Kemboi Yego, Joseph Nkurunziza, Juma Kasozi

**Affiliations:** 1 African Center of Excellence in Data Science, University of Rwanda, Kigali, Rwanda; 2 Department of Mathematics and Computer Science, Moi University, Kenya; 3 Department of Mathematics, Makerere University, Kampala, Uganda; 4 School of Economics, University of Rwanda, Kigali, Rwanda; Makerere University School of Public Health, UGANDA

## Abstract

Universal Health Coverage (UHC) is a global objective aimed at providing equitable access to essential and cost-effective healthcare services, irrespective of individuals’ financial circumstances. Despite efforts to promote UHC through health insurance programs, the uptake in Kenya remains low. This study aimed to explore the factors influencing health insurance uptake and offer insights for effective policy development and outreach programs. The study utilized machine learning techniques on data from the 2021 FinAccess Survey. Among the models examined, the Random Forest model demonstrated the highest performance with notable metrics, including a high Kappa score of 0.9273, Recall score of 0.9640, F1 score of 0.9636, and Accuracy of 0.9636. The study identified several crucial predictors of health insurance uptake, ranked in ascending order of importance by the optimal model, including poverty vulnerability, social security usage, income, education, and marital status. The results suggest that affordability is a significant barrier to health insurance uptake. The study highlights the need to address affordability challenges and implement targeted interventions to improve health insurance uptake in Kenya, thereby advancing progress towards achieving Universal Health Coverage (UHC) and ensuring universal access to quality healthcare services.

## Introduction

The goal of attaining Universal Health Coverage (UHC) has been prioritized by many nations. UHC’s objective is to provide access to essential and cost-effective healthcare to all individuals, regardless of their financial status. To achieve UHC, a secure health financing system is necessary that protects against financial risk and ensures access to necessary health services for all. In African countries like Kenya, health insurance plans have been established to achieve universal access to healthcare. These plans cover citizens who may not be able to afford necessary medical services, reducing financial barriers to care. Implementing these health insurance schemes is a crucial step towards achieving UHC and ensuring access to quality healthcare services for all. The ultimate goal of UHC is to ensure that everyone, regardless of financial situation, has access to the health services necessary for good health and well-being [1].

However, despite some strides made towards achieving the UHC goal, a significant portion of the Kenyan population still lacks access to essential healthcare services. In 2014, 60% of the population did not have access to healthcare services, and 40% were at risk of incurring catastrophic health expenditures [2]. It is reported that by 2016, only 19% of the population had any form of health cover [3]. Based on the latest FinAccess data, only 22% of surveyed individuals had either National Health Insurance Fund (NHIF) or some other form of health cover [4]. This contrasts with its counterparts like Rwanda and Ghana, which have achieved near complete UHC despite some challenges in funding [5, 6].

Nevertheless, some progress has been made in coverage over time, although universal coverage has not been achieved. For instance, Kenya introduced a free maternity policy in 2013 and expanded it to include access beyond the public sector under the Linda Mama program in 2016, which was managed by the National Hospital Insurance Fund (NHIF) [7]. Inequalities in both service coverage and financial risk protection persist, compromising quality and reach [8, 9]. Additionally, problems with governance and inequities particularly for low income populace have hindered the growth and success of the NHIF, the national social health insurance provider, which aims to provide coverage for all [10, 11].

Barriers to achieving UHC include a high proportion of the population living in poverty and unable to afford insurance premiums, a large informal sector with many uninsured individuals, high dropout rates from insurance programs, underfunding of primary healthcare, and fragmented health insurance funds [1]. Furthermore, it has been pointed out that catastrophic health expenditures are often associated with a lack of medical coverage or some form of pre-payment mechanism for risk pooling [12]. Thus, there is a need to examine the factors that influence uptake of health coverage among individuals.

Factors that influence health coverage uptake may be related to an individual’s willingness and ability to pay for coverage. Studies have shown that income level, the need for healthcare, age, marital status, enrollment in the National Social Security Fund (NSSF) and level of education are significant determinants of willingness to pay for medical coverage [13, 14]. Additionally, awareness of the services offered by the NHIF has been found to be a crucial factor in the informal sector [15]. Additionally, public-private partnerships have been suggested as a means to ease delivery of UHC [16].

Previous employment, tertiary education, and motorcycle ownership positively were also found to have significantly influenced health insurance enrollment among motorcycle taxi operators in Machakos, Kenya. However, age, household size, and membership in social welfare groups were found to have no significant effect on enrollment, which remained low at 23.3% [3]. Although the factors that influence willingness to pay for NHIF have been studied, there is still a need to investigate the factors that influence uptake among individuals who are both willing and able to pay.

This paper makes a contribution to the literature by comparing the performance of three machine learning algorithms—Random Forest, XGBoost, and Logistic Regression—in predicting health insurance uptake. It also compares the effectiveness of two data balancing techniques—random oversampling and Synthetic Minority Over-sampling Technique (SMOTE)—and compares the traditional Logistic Regression and Logistic Regression classifiers in predicting health insurance uptake. Additionally, the study utilizes these algorithms to identify the socio-economic and demographic factors that have the most significant influence on health insurance uptake. The findings of this study provide valuable insights into the most important factors that predict health insurance uptake, and can guide policymakers in designing effective health insurance policies to achieve Universal Health Coverage.

## Materials and methods

The study analyzed the 2021 FinAccess Survey by first exploring cross-tabulation. Then, it evaluated three machine learning classifiers (Random Forest, XGBoost, and logistic regression) to identify the strongest one. The selected classifier was then utilized to extract the importance of variables and make predictions.

### Data

The FinAccess Household Survey was conducted in Kenya using a cross-sectional design to gather data from individuals aged 16 and older residing in traditional households. One of the main goals of the survey was to provide data for research purposes. The survey aimed to provide estimates at the national, rural/urban, and county levels, with a sample size of 1,700 enumeration areas and 30,600 households. The sample was drawn from the Kenya Household Master Sample Frame, which is stratified into 92 sampling strata (urban and rural of the 47 counties), using a multi-stage stratified cluster sampling procedure. Enumeration areas were randomly selected from each of the 92 strata in the K-HMSF with equal probability given that the master sample was drawn with probability proportional to size.

Only individuals aged 18 and older who had national identity cards, which are essential to access formal financial services, were included in the data analysis. The survey had multiple objectives, including assessing the access, usage, and impact of financial services. Out of the 25,724 eligible individuals, 22,024 recorded successful interviews, resulting in an overall response rate of 85.6 percent. The rural dwelling response rate was 88.6 percent, while the urban response rate was 80.5 percent. The collected data was weighted and adjusted for non-responses to ensure representative data at the national and county levels. The survey provides valuable estimates at the national, rural/urban, and county levels, making it a crucial resource for policymakers, financial service providers, and researchers [4].

### Variables

The response variable in the current study is health insurance uptake, which is a binary variable representing whether an individual has taken up health insurance or not. The outcome variable Y in the study takes a binary value of either one or zero, with one indicating that an individual has taken up health insurance and zero indicating the converse.


$$Y=\left\{\begin{array}{ll} 1= & Uptake\;of\;health\;insurance/cover\\ 0= & Non-uptake\;of\;health\;cover \end{array}\right.$$ (1)

The independent variables in this study refer to a set of features that can be used to predict the likelihood of health insurance uptake. These features could include demographic information such as age, gender, income, and education level, as well as other factors such as savings usage, wealth quintile, and geographic location (whether living in rural or urban). When selecting variables to incorporate, we based our choices on prior research and relevant literature in the field, specifically [3, 13–15]. We evaluated variables that have been previously identified as significant predictors in similar research, as well as variables that hold theoretical significance for the outcome we are anticipating.

By analyzing the relationship between the response variable and the independent variables, the study aimed to identify which factors are most strongly correlated with health insurance uptake. This information can be used to develop model that predicts the likelihood of an individual taking up health insurance based on their unique set of characteristics.

### Preprocessing and feature selection

Data preprocessing involved feature selection, handling of missing values and data split. There is a need for a feature selection criterion to assess the relevance of features in determining the outcome variable and its associated categories. Some variables had a small number of missing values, these are: age group (1115 observations = 5%), dwelling tenure (70 observations = 0.3178%), meeting financial goals (2422 observations = 10.99%), and wealth quintile (70 observations = 0.3178%). To tackle this problem, we used different techniques depending on the type of analysis performed. For XGBoost and Random Forest, we utilized their inherent capabilities to handle missing values by treating them as a distinct category and training the model to capture the patterns in the data. On the other hand, for Logistic Regression analysis, we adopted the approach of row-wise dropping to handle the missing values. Since the missing values were few, the loss of information, in the latter, was minimal.

Feature selection provides benefits such as improved computational time, enhanced predictive performance, identification of relevant features, improved data quality, and saved resources in later data collection phases [17, 18]. From a dataset containing hundreds of variables, only 23 were nonredundant and relevant to the study. The excluded variables were either irrelevant, unimportant, or redundant.

The dataset was partitioned to ensure that the training data was separate from the model validation and testing processes. The train-test split ratio was set to 70:30, where the majority of the data (70%) was allocated to model training and the remaining 30% was used for validation and testing. The test-validation split was set at 1:1, resulting in a final ratio of 0.7:0.15:0.15 for training, validation, and testing, respectively. This means that 15% of the data was used for hyperparameter tuning and not utilized in testing the model’s ability to handle "unforeseen" data. It is generally recommended to use a significantly greater portion of the data for training, and in this study, a 70:30 train-test split ratio was employed [19–21].

### Exploratory data analysis

Exploratory data analysis was performed by crosstabulation and pairwise correlation analysis of the various variables with the uptake of health insurance. The cross-tabulation was used to explore the relationship between the target variable of health insurance uptake and the features, which are the features of interest. By analyzing the frequency distribution of the target variable across different values of the predictor variables, we were able to identify general patterns and correlations among the variables in the dataset. This allowed us to gauge the efficacy of the various features in modeling.

We used correlation analysis to evaluate the direction effect, and in doing so, we employed a mosaic plot to investigate the associations among the features and the outcome variable. This method allowed us to visualize the strength and direction of the relationship between each predictor and the outcome variable. Specifically, a positive correlation coefficient indicated a favorable association between the predictor and the outcome variable.

### Handling imbalanced classes

Class imbalance arises when one class, in a case of binary classification problem, has an imbalanced number of observations compared to the other class. Specifically, it occurs when the number of training data instances in one class is substantially larger than the other, resulting in a majority class and a minority class. Training a machine learning model on imbalanced data can result in biased models that perform sub-optimally compared to balanced data models [22]. However, the sampling techniques used to address class imbalance may have limitations, such as the risk of overfitting and the possibility of losing valuable information in the minority class [23, 24].

In this study, the health insurance uptake class had a significantly lower number of observations than the non-uptake class, necessitating the handling of class imbalance. To address this issue, SMOTE (Synthetic Minority Over-sampling Technique) and oversampling techniques were chosen as methods for balancing the classes. Oversampling has previously shown better performance than no oversampling strategies [25]. Nevertheless, SMOTE has shown good performance in other studies [18]. SMOTE is a technique utilized to address class imbalance in a dataset. Our study employed SMOTE to oversample the minority class. The goal was to balance the distribution of classes in the training data, as imbalanced data can negatively impact the performance of classification models. Generating additional data points using SMOTE allowed the model to better learn from the minority class and achieve improved performance [18, 26].

### Training and testing

In this study, the term "training" refers to the process of building the machine learning models using a subset of the data. This subset is used to teach the models to recognize patterns and make predictions. "Testing," on the other hand, involves evaluating the performance of the trained models on a separate subset of data that was not used during training. This allows us to assess how well the models generalize to new, unseen data [27, 28].

### Models

The purpose of the study was partly to compare the accuracy of different machine learning classifiers for predicting health insurance uptake from the three: Random Forest, XGBoost, and Logistic Regression. These classifiers were used to assess the impact of variables on predicting health insurance uptake.

### Logistic Regression

Logistic Regression traditionally, is a type of generalized linear model that involves modeling the response variable as a binary outcome with a binomial distribution and using a logit link to generalize it. Unlike the linear regression model, Logistic Regression uses a link function, with a logit link used in this study. For the set features ***X*** = *x*_1_…*x*_*n*_, the probability of pension uptake is given by:

$$Probability=E(Y|x_{1}\ldots x_{n})=\frac{exp\{\beta_{0}+\beta_{1}x_{1}+\cdots +\beta_{n}x_{n}\}}{1+exp\{\beta_{0}+\beta_{1}x_{1}+\cdots +\beta_{n}x_{n}\}}$$ (2)

where *β*_1_, ……. *β*_*n*_ are the respective estimated coefficients, and *β*_0_ is the intercept [19, 29]. However, the traditional Logistic Regression is not robust in case of missing data [30] and can effectively handle only linear correlation between independent and dependent variables [31].

Logistic Regression classifier is a method commonly used for binary classification and regression tasks, with high accuracy demonstrated in various studies, including cancer survival prediction [32] and predicting drivers of preterm birth [33]. In the latter study, Logistic Regression outperformed the decision tree classifier, with higher precision, f1 score, and area under receiver operating characteristic curve (AUC). The key advantage of Logistic Regression is that it takes the form of a non-linear regression equation, and regression-type diagnostics can be employed to evaluate the fit’s quality, relevance of explanatory variables, and the impact of individual observations on the outcome. This underscores the potential of Logistic Regression in improving prediction accuracy and enhancing decision making in various applications. However, Logistic Regression has limitations as a probability model, including the need for a specific functional relationship between variables and potential bias in probabilities due to differing proportions of observations in the sample and population.

### Random Forest

Random Forest involves using the average of multiple decision trees (also known as bagging. In Random Forest, a classification tree is generated from each new dataset, and the predictions from each tree are combined by taking the average. This results in an ensemble estimate of the classification function, represented as:

$$f_{av}\left(X\right)=\frac{1}{N}\sum_{n=1}^{N}f_{n}\left(X\right)$$ (3)

Where ƒ_n_ is the prediction obtained from training a classification tree on the n^th^ new data set [34]. Random Forest has shown to have high accuracy and robustness in claims-based Lennox-Gastaut syndrome classifier has been reliable in health insurance [35], making it a strong candidate for predicting health insurance uptake.

The Random Forest classifier is a highly effective machine learning model that offers significant advantages over traditional models. One such advantage is its ability to mitigate overfitting, which it achieves by using bagging to train multiple decision trees on diverse subsets of the input data and then aggregating their results. Another strength of the model is its capacity to handle large datasets with many features, which it handles by randomly selecting a subset of features for each tree. Additionally, the Random Forest classifier excels at modeling complex nonlinear relationships between input variables and the target variable and can easily handle datasets with missing data by utilizing surrogate splits. Finally, the Random Forest model is highly user-friendly and intuitive, requiring minimal parameter tuning and providing a clear visualization of the relative importance of each variable in the input data [36, 37].

### XGBoost

The XGBoost algorithm uses the boosting method to combine multiple decision trees. It begins by making the same initial predictions for all samples. Then, it builds a decision tree by examining the residuals of each sample in the training set and identifying the partition with the greatest gain. The gain is determined by adding up the similarity scores of the child nodes and subtracting the parent node’s similarity score, and the threshold in decision nodes, serves as the basis for splitting. The trees are pruned, and the output value of each node is calculated in log-odds. Finally, the predicted probabilities are refined by repeatedly iterating this process and converting the log-odds back into probabilities [37].

### Model performance metrics

Kappa score is a measure of inter-annotator agreement that takes into account the agreement expected by chance. A kappa score of 1.0 indicates perfect agreement, whereas a score close to 0 indicates random agreement. Recall score is a measure of the ability of a model to identify positive cases. In imbalanced datasets, recall score is an important metric, as it indicates how well the model identifies the minority class. F1 score is the harmonic mean of precision and recall, and provides a single score that balances precision and recall. Accuracy is the proportion of correct predictions made by the model, but in imbalanced datasets, accuracy can be misleading, as the model may achieve high accuracy by simply predicting the majority class all the time [38, 39]

The AUC, or area under the receiver operating characteristic curve, is a metric used to evaluate the performance of a classifier in distinguishing positive and negative classes. It plots the true positive rate against the false positive rate for every possible threshold and ranges from 0 to 1. In this study, AUC scores were used to evaluate the ability of classifiers to predict health insurance uptake based on socio-economic and demographic factors. Confusion matrices were also used to evaluate the performance of the models by comparing predicted labels to true labels of the test data.

### Limitation of machine learning

It is important to note that machine learning models have limitation in terms of interpretation [40]. To address this limitation, a distribution of predicted probabilities across the identified important factors was examined. This approach provides insight into the relationship between these factors and the likelihood of health insurance uptake, enhancing the interpretability of the results. The use of predicted probabilities has been done in other studies [39, 41, 42]

### Selection of the optimal model

The selection of the best model involved evaluating several performance metrics, including accuracy, Kappa score, F1 score, recall score, and precision score. When selecting the best model, it is important to consider a combination of these metrics, taking into account the specific goals and requirements of the study. For example, when the focus is on correctly identifying positive cases (health insurance uptake) to ensure comprehensive coverage, a higher recall score would be desirable. On the other hand, when avoiding false positives is a priority, precision score becomes more important. Ultimately, the best model selection should align with the specific objectives and trade-offs that need to be considered in the context of the study.

## Results

### Results from exploratory analysis

Table 1 presents a cross-tabulation that displays the relationship between health insurance uptake and various socio-economic and demographic factors, including gender, marital status, cluster type, savings usage, mobile ownership, NSSF usage, financial health, loan/debt default, ability to cope with risk, age groups, tenure status, ability to invest in livelihoods, and wealth quintile. The following key insights can be gleaned from the table:

**Table 1:** The distribution of health insurance based on various factors.

Variable	Level	No	Yes	Total
Selected Respondent Gender	Male	7103(76.1%)	2236(23.9%)	9339 (100.0%)
	Female	10264(80.9%)	2421(19.1%)	12685(100.0%)
Marital status of Respondent	Single/Never Married	4903(84.5%)	898(15.5%)	5801(100.0%)
	Divorced/separated	1301(80.7%)	311(19.3%)	1612(100.0%)
	Widowed	2101(81.4%)	481(18.6%)	2582(100.0%)
	Married/Living with partner	9045(75.3%)	2959(24.7%)	12004(100.0%)
	Other	17(68.0%)	8(32.0%)	25(100.0%)
Cluster Type (rural/urban)	Rural	11987(82.9%)	2468(17.1%)	14455(100.0%)
	Urban	5380(71.1%)	2189(28.9%)	7569(100.0%)
Savings Usage overall	Currently have	11767 (74.9%)	3953(25.1%)	15720(100.0%)
	Used to have	1509 (84.9%)	268(15.1%)	1777(100.0%)
	Never had	4091(90.4%)	436(9.6%)	4527(100.0%)
Mobile Ownership	No	4092(94.7%)	228(5.3%)	4320(100.0%)
	Yes	13275(75.0%)	4429(25.0%)	17704(100.0%)
NSSF usage	Currently have	184(10.7%)	1542(89.3%)	1726(100.0%)
	Used to have	527(63.0%)	309(37.0%)	836(100.0%)
	Never had	16656(85.6%)	2806(14.4%)	19462(100.0%)
Financially healthy	No	15346(83.4%)	3057(16.6%)	18403(100.0%)
	Yes	2021(55.8%)	1600(44.2%)	3621(100.0%)
Defaulted on Loans/Debt	No	11574(79.20%)	3039(20.80%)	14613(100%)
	Yes	5793(78.17%)	1618(21.83%)	7411(100%)
Adults with ability to cope with risk	No	14329(84.1%)	2718(15.9%)	17047(100.0%)
	Yes	3038(61.0%)	1939(39.0%)	4977(100.0%)
Experienced any shock	Did not experience	5133(79.9%)	1292(20.1%)	6425(100.0%)
	Experienced shock	12234(78.4%)	3365(21.6%)	15599(100.0%)
Age groups	18–25 yrs	3864(88.2%)	518(11.8%)	4382(100.0%)
	26–64 yrs	10638(75.1%)	3528(24.9%)	14166(100.0%)
	65+ yrs	1797(76.1%)	564(23.9%)	2361(100.0%)
Tenure Status of Main Dwelling Unit	Purchased	93(71.5%)	37(28.5%)	130(100.0%)
	Constructed	12582(82.4%)	2684(17.6%)	15266(100.0%)
	Inherited	723(77.8%)	206(22.2%)	929(100.0%)
	Rented	3918(69.6%)	1711(30.4%)	5629(100.0%)
Adults with ability to invest in livelihoods	No	11102(83.3%)	2233(16.7%)	13335(100.0%)
	Yes	6265(72.1%)	2424(27.9%)	8689(100.0%)
wealth Index	Lowest 40%	8144(92.7%)	638(7.3%)	8782(100.0%)
	Middle 40%	6542(74.5%)	2240(25.5%)	8782(100.0%)
	Richest 20%	2630(59.9%)	1760(40.1%)	4390(100.0%)
Banked	No	5267(76.2%)	1649(23.8%)	6916(100.0%)
	Yes	12100(80.1%)	3008(19.9%)	15108(100.0%)
Education level of Respondent	None	3659(91.8%)	328(8.2%)	3987(100.0%)
	Primary	7424(83.9%)	1422(16.1%)	8846(100.0%)
	Secondary	5109(77.0%)	1522(23.0%)	6631(100.0%)
	Tertiary	1160(45.8%)	1375(54.2%)	2535(100.0%)
	Other	15(60.0%)	10(40.0%)	25(100.0%)
Investment score of Respondent	Score 0	4171(87.5%)	594(12.5%)	4765(100.0%)
	Score 1	6931(80.9%)	1639(19.1%)	8570(100.0%)
	Score 2	4602(75.0%)	1535(25.0%)	6137(100.0%)
	Score 3	1663(65.2%)	889(34.8%)	2552(100.0%)

Gender: According to the data, a higher proportion of females (80.9%) did not take up health insurance compared to males (76.1%). Marital status: Single/never married respondents had the highest proportion of non-health-insurance-takers (84.5%), followed by widowed (81.4%) and married/living with a partner (75.3%). Cluster Type (Rural/Urban): A higher proportion of rural respondents (82.9%) did not take up health insurance compared to urban respondents (71.1%). Savings Usage: Respondents who had never had savings were less likely to take up health insurance (90.4%) compared to those who currently have savings (74.9%) and those who used to have savings (84.9%). Mobile Ownership: The data shows that a higher proportion of respondents who do not own a mobile phone (94.7%) did not take up health insurance compared to those who own a mobile phone (75.0%). NSSF Usage: The data shows that a higher proportion of respondents who have never used the National Social Security Fund (NSSF) (85.6%) did not take up health insurance compared to those who currently use NSSF (10.7%) and those who used to use NSSF (63.0%). Financially healthy: The data shows that a higher proportion of respondents who are not financially healthy (83.4%) did not take up health insurance compared to those who are financially healthy (55.8%). Defaulted on Loans/Debt: The data shows that a higher proportion of respondents who have defaulted on loans or debt (78.17%) did not take up health insurance compared to those who have not defaulted (79.20%).

Adults with ability to cope with risk: The data shows that a higher proportion of respondents who do not have the ability to cope with risk (84.1%) did not take up health insurance compared to those who have the ability to cope with risk (61.0%). Experienced any shock: The data shows that a higher proportion of respondents who have experienced a shock (78.4%) did not take up health insurance compared to those who have not experienced a shock (79.9%). Age groups: The data shows that a higher proportion of older adults (76.1%) did not take up health insurance compared to younger adults (88.2%). Tenure status of main dwelling unit: The data shows that a higher proportion of respondents who rent their main dwelling unit (69.6%) did not take up health insurance compared to those who have purchased (71.5%), constructed (82.4%) or inherited (77.8%) their main dwelling unit. Adults with ability to invest in livelihoods: The data shows that a higher proportion of respondents who do not have the ability to invest in livelihoods (83.3%) did not take up health insurance compared to those who have the ability to invest in livelihoods (72.1%). Wealth Index: The data shows that a higher proportion of respondents in the lowest 40% of the new wealth index (92.7%) did not take up health insurance compared to those in the middle 40% (74.5%) and the richest 20% (71.3%) index.

### Model metric results on imbalanced data

The three machine learning models were trained initially on imbalanced data. Table 2 shows the performance of the three models upon training on the imbalanced data. The results are from the test set. The performance metrics of the models trained on imbalanced data showed that the Random Forest model performed the best overall with a Kappa score of 0.5922, Recall score of 0.7550, F1 score of 0.7941, and Accuracy of 0.8881. The Logistic Regression model also performed relatively well, with a Kappa score of 0.5574, Recall score of 0.7366, F1 score of 0.7761, and Accuracy of 0.8803. Finally, the XGBoost model had the lowest Kappa score of 0.5584, Recall score of 0.7461, F1 score of 0.7777, and Accuracy of 0.8760. Nevertheless, the difference in Kappa score between XGBoost (0.5584) and Random Forest (0.5922) was not substantial, and neither model had a Kappa score exceeding 0.5922, demonstrating that the models performed inadequately on the imbalanced data, as anticipated.

**Table 2:** Model performance metrics on imbalanced dataset.

	Model	Kappa score	Recall score	F1 score	Accuracy
1	Logistic Regression	0.5574	0.7366	0.7761	0.8803
2	Random Forest	0.5922	0.7550	0.7941	0.8881
3	XGBoost	0.5584	0.7461	0.7777	0.8760

### Model metric results on balanced data upon oversampling

Table 3 shows the performance metrics of each model when the data is balanced by oversampling. Random Forest and XGBoost performed better than Logistic Regression. The Logistic Regression model had a Kappa score of 0.5364, Recall score of 0.7676, F1 score of 0.7677, and Accuracy of 0.7689. The Random Forest model had a significantly higher Kappa score of 0.9273, Recall score of 0.9640, F1 score of 0.9636, and Accuracy of 0.9636.

**Table 3:** Model metrics trained on data balanced by oversampling.

	Model	Kappa score	Recall score	F1 score	Accuracy
1	Logistic Regression	0.5364	0.7676	0.7677	0.7689
2	Random Forest	0.9273	0.9640	0.9636	0.9636
3	XGBoost	0.7558	0.8781	0.8779	0.8779

The XGBoost model had a Kappa score of 0.7558, Recall score of 0.8781, F1 score of 0.8779, and Accuracy of 0.8779. Based on these results, both Random Forest performed the most compared to XGBoost and Logistic Regression when using balanced data oversampling. Overall, the Random Forest model performed the best with the highest Kappa, Recall, F1, and Accuracy scores.

### Model metric results upon using SMOTE

Table 4 shows the performance metrics of each model when the data is balanced by SMOTE.

**Table 4:** Model metrics upon balancing using SMOTE.

	Model	Kappa score	Recall score	F1 score	Accuracy
1	Logistic Regression	0.5264	0.7624	0.7625	0.7642
2	Random Forest	0.8038	0.9015	0.9019	0.9021
3	XGBoost	0.7999	0.8991	0.8998	0.9003

The results show that using the SMOTE method of balancing the data had a positive impact on the performance of the models compared to using unbalanced data. The results indicate that the Random Forest model performed the best, with a kappa score of 0.8038, recall score of 0.9015, F1 score of 0.9019, and accuracy of 0.9021. The XGBoost model also performed well, with a kappa score of 0.7999, recall score of 0.8991, F1 score of 0.8998, and accuracy of 0.9003. The Logistic Regression model had the lowest performance metrics among the three models. Both Random and XGBoost performed better with SMOTE than without, with Random Forest having a Kappa score of 0.8038, which is higher than its score in the unbalanced data, and XGBoost having a Kappa score of 0.7999, also higher than its score in the unbalanced data. In conclusion, using SMOTE as a method of balancing the data had a positive impact on the models’ performance, and Random Forest was the best performing model in terms of Kappa, recall, F1, and accuracy scores.

### Confusion matrices

**Fig 1:**
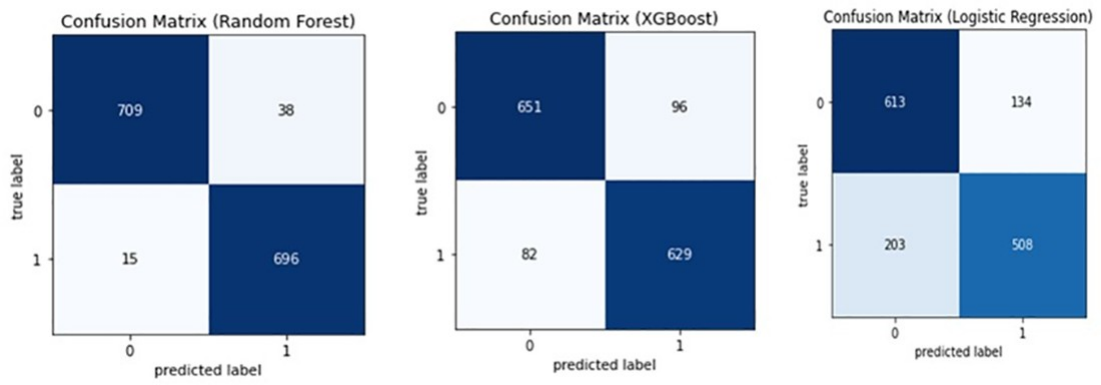
Confusion matrices.

Fig 1 displays confusion matrices for the three models: Random Forest, XGBoost and Logistic Regression.

The Random Forest model achieved the highest accuracy rate, accurately predicting 709 positive instances and 696 negative instances, resulting in a high true positive rate and true negative rate. Although it misclassified 38 negative instances as positive and 15 positive instances as negative, the model still had an overall high performance. The XGBoost model had a lower overall performance compared to the Random Forest model, with a false positive rate of 0.132 and a false negative rate of 0.115. Its true positive rate and true negative rate were also lower than the Random Forest model. The Logistic Regression model had the poorest performance, with the highest false positive rate and false negative rate, resulting in a lower true positive rate and true negative rate than the other models. Based on the confusion matrices provided, the Random Forest model achieved the best performance, followed by the XGBoost model and the Logistic Regression model.

### Areas under receiver operating characteristic curve (AUC)

**Fig 2:**
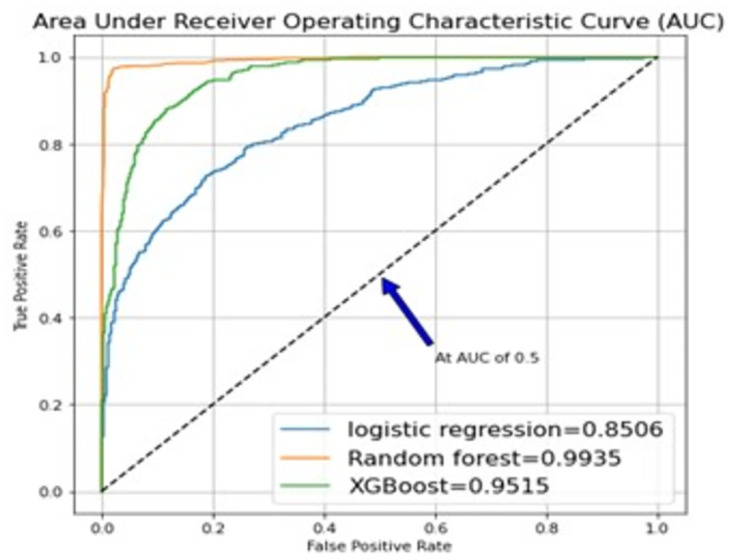
The AUCs for the models.

The model performances were evaluated using the area under the operating characteristic curve (AUC) as displayed in Fig 2.

The AUC measures the ability of a model to distinguish between positive and negative classes. AUC scores closer to 1 indicate better model performance. In this case, Random Forest had the highest AUC score of 0.9928, followed by XGBoost with 0.9367, and Logistic Regression with 0.8567. These results suggest that Random Forest had the best ability to distinguish between positive and negative classes, while Logistic Regression had the lowest ability. Based on these results, Random Forest had the best model performance in terms of AUC and can be considered as a good candidate for predicting health insurance uptake.

### Variable importance from each algorithm

The feature importance ranking in a Random Forest model gives a measure of the impact that each feature has on the target variable (in this case, health insurance uptake).

### Variable importance from Random Forest

**Fig 3:**
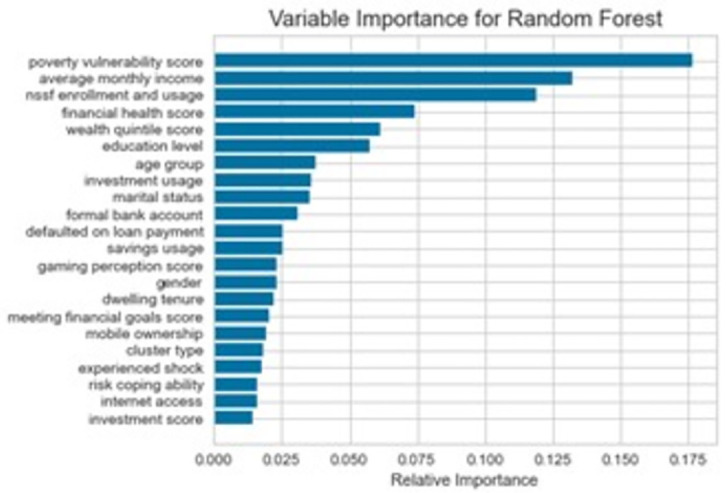
Variable importance from the Random Forest model.

Fig 3 displays the extraction of variable importance from the Random Forest model, which was found to be the most robust.

The findings show that the Poverty Vulnerability Score, Average Monthly Income, and NSSF Enrollment and Usage are the three most important factors that influence health insurance uptake. Financial stability, education level, age group, and investment usage are also important factors, while factors such as gender, gaming perception score, dwelling tenure, and cluster type have relatively low importance. These findings can help inform targeted interventions to increase health insurance uptake among vulnerable populations.

### Variable importance from XGBoost

**Fig 4:**
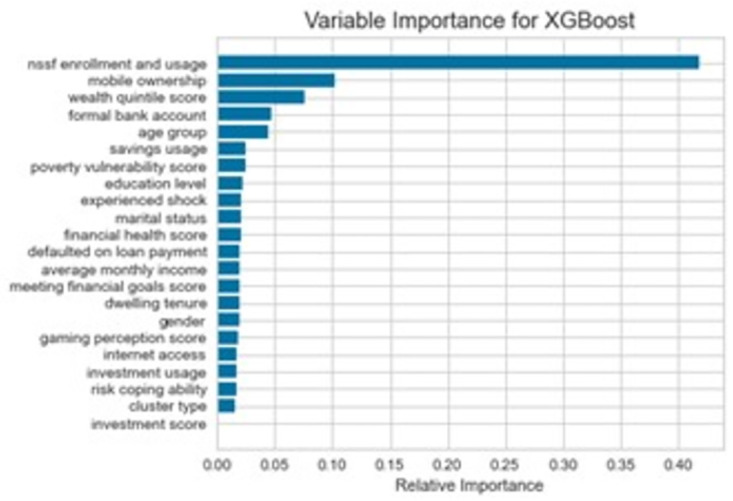
Variable importance from the XGBoost model.

Fig 4 displays feature importance of XGBoost mode. Based on the figure, the most important feature as NSSF (National Social Security Fund) Enrollment and Usage, which had an importance score of 0.418172. This suggests that having a social security fund, such as NSSF, may play a significant role in an individual’s decision to enroll in health insurance. Mobile ownership was the second most important feature with an importance score of 0.10154, indicating that owning a mobile phone may be a factor that influences health insurance uptake. Other features that were found to be important in predicting health insurance uptake include wealth quintile score, formal bank account, age group, and savings usage.

### Variable importance from Logistic Regression classifier

**Fig 5:**
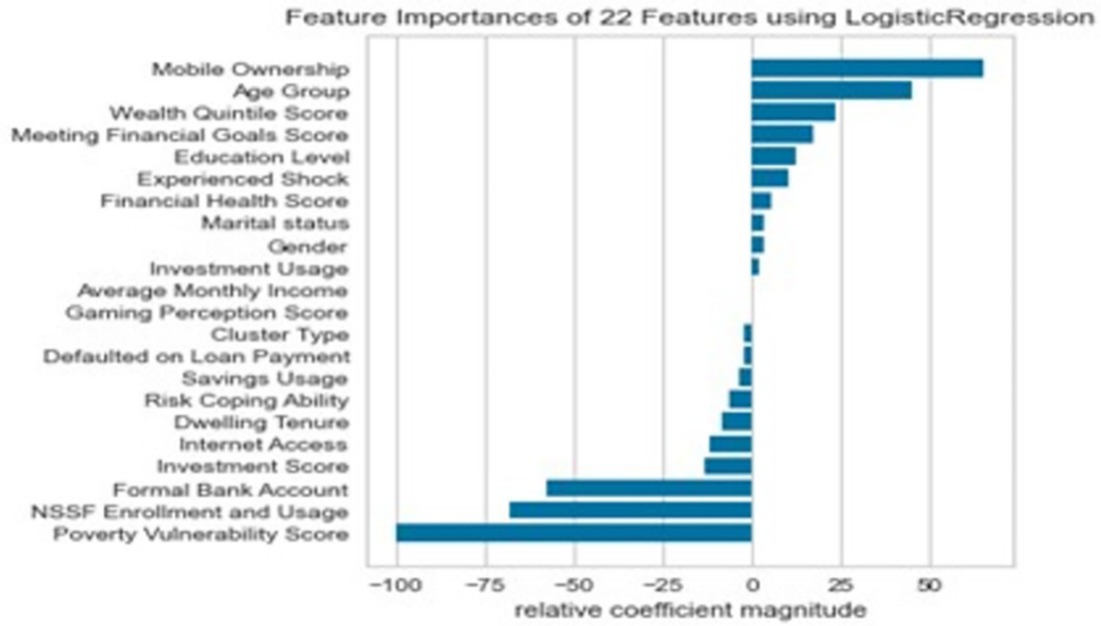
Variable importance from the Logistic Regression classifier.

The Logistic Regression classifier analysis in Fig 5 highlights the significant socio-economic and demographic factors that influence health insurance uptake. The advantage of variable importance from Logistic Regression classifier over the ones extracted from Random Forest and XGBoost is the ability to give the direction of relationship apart from the relative magnitude. Mobile ownership emerged as the most important feature, indicating that individuals who own a mobile phone are more likely to uptake health insurance. Age group, wealth quintile score, and meeting financial goals score were also important factors, implying that older individuals, those with higher wealth quintile scores, and those who are better at meeting their financial goals are more likely to uptake health insurance. Education level, experienced shock, financial health score, and marital status were also found to be important determinants of health insurance uptake.

On the other hand, some features had a negative impact on health insurance uptake. Lack of NSSF enrollment and usage, lack of formal bank account, and poverty vulnerability score were found to have a negative influence on health insurance uptake. This suggests that individuals who are not enrolled in NSSF, are unbanked, or have a high poverty vulnerability score are less likely to uptake health insurance.

## Discussion

### Model selected and the data sampling

The results of the study examining the prediction of health insurance uptake using Random Forest, XGBoost and Logistic Regression, indicate that the use of balanced data improved the performance of the models. The evaluation metrics, including Kappa score, recall, F1 score, accuracy, and AUC, demonstrate that Random Forest yielded the most optimal results. This highlights the difficulties of modelling imbalanced datasets [43].

The application of both random oversampling and SMOTE data balancing techniques led to a positive effect on the models’ performance, with Random Forest exhibiting superior results compared to the other models. Although XGBoost performed well in terms of balanced error measures and overall accuracy, it did not outperform Random Forest. Conversely, Logistic Regression showed subpar performance compared to Random Forest and XGBoost. This is consistent with other studies where Random Forest outperformed the other models [35]. Based on socio-economic and demographic factors in similar datasets or conditions, Random Forest could therefore be deemed a suitable model for predicting health insurance uptake.

### Feature discussion

**Fig 6:**
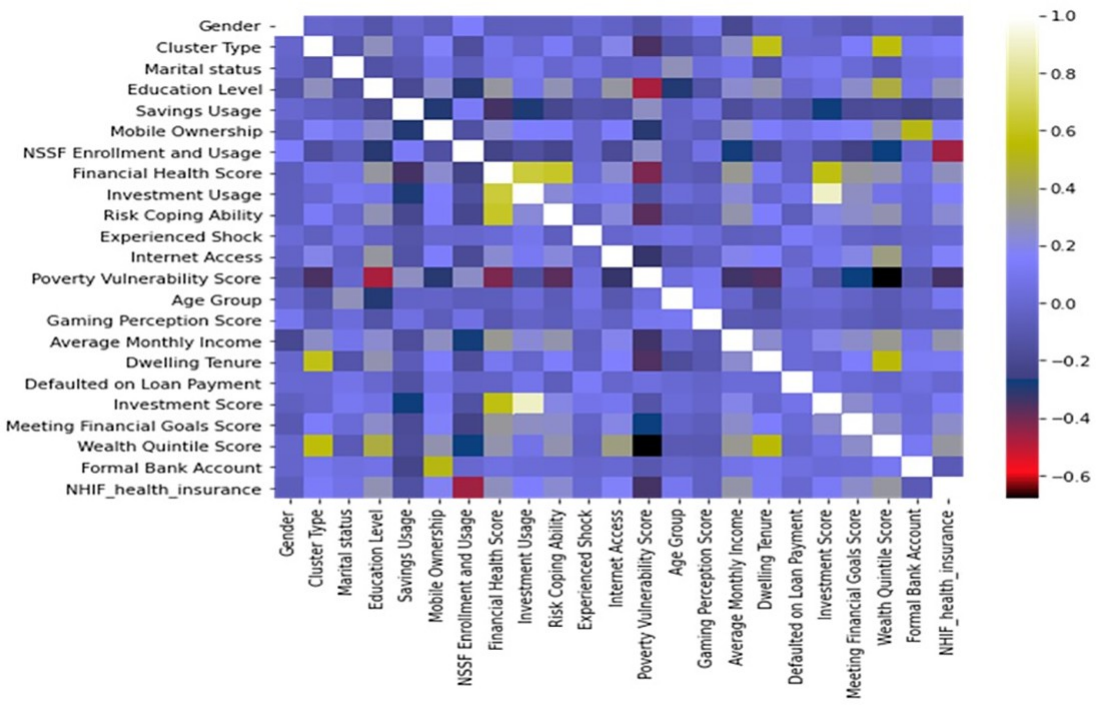
Mosaic plot.

The results from cross-tabulation, the mosaic plot in Fig 6 and the variable importance extracted from the tree models point to ongoing inequities in the levels of health insurance uptake. The wealthier classes of the population are found to have a higher likelihood of health insurance uptake, suggesting affordability as the main issue hindering the health uptake. These findings are consistent with prior observations of pro-wealthy income-related disparities in health insurance coverage. In a contributory and voluntary system, individuals who are financially constrained may not be able to pay the required premiums. Therefore, subsidizing premium payments for these individuals is necessary [9]. Despite the funding challenges, demand-side financing mechanisms should be a priority in the public-private partnerships the government is seeking to establish. However, the benefits of these mechanisms will not be fully realized unless the infrastructure for delivering primary health services is strong [44].

**Fig 7:**
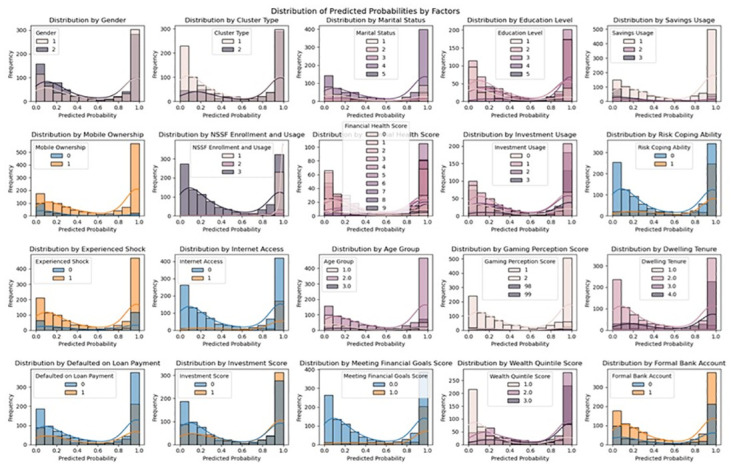
Distribution plots of predicted probabilities.

The distribution plots in Fig 7 show the distribution of predicted probabilities for each factor category. In gender categories, there is a higher likelihood of uptake among men compared to women. However, some improvement has been noted in the disparity between genders in the levels of health insurance uptake. According to the 2016 FinAccess survey, 24.59% of men and 15.09% of women had health insurance. In the 2021 survey, the figures were 23.9% for men and 19.1% for women. Despite some improvement in reducing the gender gap in health insurance uptake, there remains a slight disparity between males and females. The progress can be attributed to the revitalization of specific programs, such as the Linda Mama program, aimed at increasing health insurance uptake among women. Consequently, it is imperative to continue investing in and supporting such programs to further narrow the gender gap and promote equitable access to health insurance [7, 45].

Moreover, a relationship was observed between higher levels of education and higher rates of health insurance uptake. According to the distribution plots shown in the predicted probability distribution across different education categories, it indicated that probability of health insurance uptake increases with education level, with the highest probability uptake observed among those with tertiary education, followed by those with secondary education, primary education and no education. This finding slightly differs from a previous study by Orangi et al. (2021), which observed the highest rates of uptake among those with secondary education, but is consistent with other studies that have found an increase in levels of health insurance uptake with higher levels of education [3]. Therefore, the results here suggest that education level may impact the likelihood of health insurance uptake. To maximize health insurance uptake and ultimately achieve Universal Health Coverage, the implementation of educational programs and policymaking is necessary. It is suggested to establish partnerships with the National Hospital Insurance Fund, private health insurance providers, industry associations, and regulatory bodies as a strategy.

The analysis of the variable importance indicated that marital status was a crucial predictor of health insurance uptake. The distribution plots show the predicted probability distribution across different marital status. The plot shows differences in the predicted probabilities of health insurance uptake based on marital status. Those who were either married or living with a partner had the highest health insurance uptake likelihood, followed by those who were divorced or separated, widowed, and single or never married. One possible reason for this pattern could be the financial difficulties faced by individuals who are divorced, separated, widowed, or single/never married, which affects their ability to pay for health insurance. Alternatively, it could be due to the single payment option provided by some health insurance schemes, where both partners are insured when one is covered. This finding aligns with Kimani et al. (2012), who emphasized the advantage of being married or living with a partner, who have a dual-income household and financial support.

### Comparison of the traditional Logistic Regression model with the Logistic Regression classifier

Results from traditional Logistic Regression analysis was compared with those from Logistic Regression classifier. There were some similarities and differences between the important variables identified by the Logistic Regression model and the Logistic Regression classifier. Both models identify age group, wealth quintile level, meeting own financial goals, education level, marital status, ownership of a mobile phone, and financial health score as significant predictors of health insurance uptake. However, the Logistic Regression classifier identifies poverty vulnerability as the most important negative predictor of health insurance uptake, while the traditional Logistic Regression model did not include this variable in the list of significant predictors.

One possible reason for these differences could be the distinct approaches used by the two models. The Logistic Regression model utilizes a linear relationship between the predictor variables and the outcome variable, and examines the statistical significance of each coefficient by testing their p-values for significance. However, the use of p-values has encountered some setbacks [46]. This approach presumes that the relationship between the predictor variables and the outcome variable is both linear and additive, which may not always be the case in the analyzed data [31].

On the other hand, the Logistic Regression classifier does not assume linearity to model the relationship between the predictor variables and the outcome variable. This approach is more flexible and can capture non-linear relationships between the variables. However, it can also be prone to overfitting if the model is too complex or if there are too many predictor variables. Relative importance values measure the contribution of each variable to the overall prediction accuracy of the model, while p-values test the statistical significance of each coefficient in the model.

In summary, the differences between the important variables identified by the traditional Logistic Regression model and the Logistic Regression classifier may be due to the different modeling approaches and statistical tests used by the two models. It is important to consider these differences and limitations when interpreting the results and drawing conclusions about the predictors of health insurance uptake.

## Conclusions and recommendations

The study aimed to use machine learning to predict health insurance uptake and inform policymaking. The results showed that Random Forest performed best among the models (Random Forest, XGBoost, and Logistic Regression) in terms of evaluation metrics such as Kappa score, recall, F1 score, accuracy, and AUC. The use of oversampling balancing techniques had a positive effect on the models’ performance. The results indicate that socio-economic and demographic variables, including education and marital status, play a significant role in health insurance uptake. Wealthier classes of the population and those with higher education levels had a higher likelihood of health insurance uptake, suggesting affordability as the main issue hindering uptake. Gender disparity was found to be not as pronounced as some other factors. The results suggest that demand-side financing mechanisms, educational programs, and partnerships with health insurance providers should be prioritized to achieve Universal Health Coverage.

## Supporting information

S1 Appendix (DOCX)

S1 Data (XLSX)

S1 File (PDF)
