# Supplementary material for: Predicting health insurance uptake in Kenya using Random Forest: An analysis of socio-economic and demographic factors
Source: PLoS One. 2023 Nov 30;18(11):e0294166. doi: 10.1371/journal.pone.0294166 (PMC10688734; doi:10.1371/journal.pone.0294166)
Supplement: S1 Appendix — (DOCX) [file pone.0294166.s001.docx]

# Appendices

## Appendix 1: Table 5: Explanation of the features and their levels of measurement.

| Feature | Explanation | Range of Values/Levels of measurement |
| --- | --- | --- |
| NSSF usage | NSSF usage | Currently have, Never had, Used to have |
| Monthly income | Average monthly income in currency value (KES) | In currency values |
| Formal Bank Account | Having a formal bank account | Yes, No |
| Poverty vulnerability | Poverty vulnerability index | 0 to 1 (continuous) |
| Education level | Highest level of education level attained | None, Primary, Secondary, Tertiary, Other |
| Age group | Age group of the respondent | 18-25 yrs, 26-64 yrs, 65+ yrs |
| Internet access | Can access internet | No, yes, refused to answer |
| Financial health score | Financially healthy | Yes, No |
| Wealth quintile | Wealth quintile level | Lowest, Middle, Richest |
| Dwelling tenure | tenure place of dwelling residence | Purchased, Constructed, Inherited, Rented |
| Marital status | Marital status | Married/living with partner, Single/never married, Widowed |
| Savings usage | Making savings | Currently have, used to have, never had |
| Gender | Gender of the respondent | Male, Female |
| Meeting financial goals | Able to meet own financial goals | Yes, No |
| Cluster type (rural/urban) | Nature of residence (rural/urban) | Rural, urban |
| Mobile ownership | Owning a mobile phone | Yes, No |
| Gaming perception | Perception toward gaming | Positive, Negative |
| Experienced any shock | Experienced any shock | Did not experience, Experienced shock |
| Investment score | Adults with ability to invest in livelihoods | No, Yes |
| Investment usage | Usage of investment | Score (0,1,2,3) |
| Risk cope | Able to cope with risk | No, Yes |

## Appendix 2: Table 6: Results from Traditional Logistic Regression

| Dep. Variable | Health insurance uptake | No. Observations | 18697 |
| --- | --- | --- | --- |
| Model | Logit | Df Residuals | 18675 |
| Method | MLE | Df Model | 21 |
| Date | Sun, 09 Apr 2023 | Pseudo R-squ. | 0.3205 |
|  |  | 14 Log-Likelihood | -6704.7 |
| converged | True | LL-Null | -9867.6 |
|  |  | LLR p-value | 0 |

|  | **Coefficients** | **std err** | **z** | **P>\|z** | **[0.025** | **0.975]** |
| --- | --- | --- | --- | --- | --- | --- |
| Gender | 0.0033 | 0.045 | 0.073 | 0.942 | -0.085 | 0.092 |
| Cluster Type | -0.112 | 0.06 | -1.862 | 0.063 | -0.23 | 0.006 |
| Marital status | 0.0679 | 0.019 | 3.497 | 0 | 0.03 | 0.106 |
| Education Level | 0.2199 | 0.028 | 7.851 | 0 | 0.165 | 0.275 |
| Savings Usage | -0.0874 | 0.034 | -2.541 | 0.011 | -0.155 | -0.02 |
| Mobile Ownership | 1.2317 | 0.092 | 13.396 | 0 | 1.051 | 1.412 |
| NSSF Enrollment and Usage | -1.3997 | 0.035 | -40.011 | 0 | -1.468 | -1.331 |
| Financial Health Score | 0.069 | 0.022 | 3.177 | 0.001 | 0.026 | 0.112 |
| Investment Usage | 0.0544 | 0.054 | 1.008 | 0.314 | -0.051 | 0.16 |
| Risk Coping Ability | 0.0069 | 0.072 | 0.096 | 0.924 | -0.134 | 0.148 |
| Experienced Shock | 0.0933 | 0.057 | 1.643 | 0.1 | -0.018 | 0.205 |
| Internet Access | -0.1845 | 0.062 | -2.98 | 0.003 | -0.306 | -0.063 |
| Poverty Vulnerability Score | -2.1954 | 0.151 | -14.583 | 0 | -2.49 | -1.9 |
| Age Group | 0.6787 | 0.043 | 15.943 | 0 | 0.595 | 0.762 |
| Gaming Perception Score | -0.0006 | 0.001 | -0.6 | 0.548 | -0.003 | 0.001 |
| Average Monthly Income | 2.47E-05 | 2.54E-06 | 9.75 | 0 | 1.98E-05 | 2.97E-05 |
| Dwelling Tenure | -0.1197 | 0.031 | -3.831 | 0 | -0.181 | -0.058 |
| Defaulted on Loan Payment | 0.0018 | 0.047 | 0.039 | 0.969 | -0.09 | 0.094 |
| Investment Score | -0.246 | 0.09 | -2.746 | 0.006 | -0.422 | -0.07 |
| Meeting Financial Goals Score | 0.3734 | 0.058 | 6.39 | 0 | 0.259 | 0.488 |
| Wealth Quintile Score | 0.3743 | 0.047 | 7.957 | 0 | 0.282 | 0.467 |
| Formal Bank Account | -1.1151 | 0.055 | -20.397 | 0 | -1.222 | -1.008 |
| Meeting Financial Goals | 0.3734 | 0.058 | 6.39 | 0 | 0.259 | 0.488 |
| Wealth Quintile Score | 0.3743 | 0.047 | 7.957 | 0 | 0.282 | 0.467 |
| Formal Bank Account | -1.1151 | 0.055 | -20.397 | 0 | -1.222 | -1.008 |

A “traditional” Logistic Regression model was also used to analyze the factors influencing health insurance uptake and later compared with Logistic Regression classifier. The model explains a substantial proportion of the variation in health insurance uptake, with a pseudo-R-squared value of 0.3205, although there may be other important factors not included in the analysis.

Gender did not have a significant effect on health insurance uptake, as indicated by the coefficient estimate of 0.0033 and the p-value of 0.942. However, other covariates had significant effects on health insurance uptake, such as marital status, education level, savings usage, mobile ownership, financial health score, internet access, poverty vulnerability score, age group, investment score, meeting financial goals score, and wealth quintile score. For example, individuals who have enrolled in NSSF and used it have a significantly lower likelihood of taking up health insurance, as indicated by the coefficient estimate of -1.3997 and the p-value of 0. The negative coefficient suggests that individuals who have enrolled in NSSF and used it are less likely to take up health insurance, all other things being equal. In contrast, having a formal bank account has a significant positive effect on health insurance uptake, as indicated by the coefficient estimate of -1.1151 and the p-value of 0. The Logistic Regression model also analyzed the factors influencing other financial behaviors, such as investment usage, risk coping ability, and defaulted loan payments. However, these variables did not have statistically significant effects on the outcome variable.

Overall, the results suggest that various demographic, economic, and financial factors are associated with the likelihood of taking up health insurance, and policymakers and insurers may use these insights to develop strategies to increase insurance uptake among different groups of individuals. It's important to keep in mind the limitations and assumptions of Logistic Regression models when interpreting these results.
