# Supplementary material for: Predicting health insurance uptake in Kenya using Random Forest: An analysis of socio-economic and demographic factors
Source: PLoS One. 2023 Nov 30;18(11):e0294166. doi: 10.1371/journal.pone.0294166 (PMC10688734; doi:10.1371/journal.pone.0294166)
Supplement: S1 File — (PDF) [file pone.0294166.s003.pdf]

Import Libraries

In [2]:
```
#import scipy
import pandas as pd
import matplotlib
import matplotlib.pyplot as plt
import seaborn as sns
import numpy as np
import warnings
from sklearn.model_selection import cross_val_score
from mlxtend.plotting import plot_decision_regions, plot_confusion_matrix
from sklearn.preprocessing import StandardScaler,FunctionTransformer
pd.options.display.max_columns = None # Remove pandas display column number li
#InteractiveShell.ast_node_interactivity = "all" # Display all values of a jupy
import sys
from sklearn.metrics import (precision_score, recall_score,
                             f1_score)
from sklearn.metrics import mean_absolute_error
from sklearn.calibration import CalibratedClassifierCV, calibration_curve
from sklearn.model_selection import train_test_split
import warnings
warnings.filterwarnings(action='ignore')
#command for displaying visualizations within the notebook
%matplotlib inline
from sklearn.ensemble import RandomForestClassifier, VotingClassifier
from sklearn.preprocessing import RobustScaler
from sklearn.metrics import confusion_matrix, recall_score, precision_score,f1_
from sklearn.model_selection import learning_curve, GridSearchCV
from sklearn.metrics import roc_curve, auc, confusion_matrix, classification_r
from sklearn.linear_model import LogisticRegression
#from sklearn.grid_search import GridSearchCV
from sklearn.model_selection import learning_curve
from sklearn.model_selection import KFold
from sklearn.ensemble import GradientBoostingClassifier, RandomForestClassifie
from sklearn import preprocessing, metrics
import warnings
warnings.filterwarnings('ignore') # to supress warnings
from sklearn.model_selection import learning_curve, GridSearchCV
#from sklearn.cross_validation import train_test_split
from sklearn.model_selection import learning_curve, GridSearchCV
from sklearn.model_selection import train_test_split
from sklearn.metrics import roc_curve, auc, confusion_matrix, classification_r
from sklearn.linear_model import LogisticRegression
from sklearn.model_selection import learning_curve
from sklearn.model_selection import KFold
from sklearn.ensemble import GradientBoostingClassifier, RandomForestClassifie
from sklearn import preprocessing, metrics
from sklearn.model_selection import StratifiedKFold
warnings.filterwarnings('ignore') # to supress warnings
import matplotlib.transforms as mtransforms
import matplotlib.lines as mlines
%matplotlib inline
from matplotlib.pylab import rcParams
rcParams['figure.figsize'] = 12, 4
import random
random.seed(2)
from xgboost import XGBClassifier
```

```
from sklearn.preprocessing import LabelEncoder
```

```
C:\Users\Francis Yego\anaconda3\lib\site-packages\numpy\_distributor_init.py:
30: UserWarning: loaded more than 1 DLL from .libs:
C:\Users\Francis Yego\anaconda3\lib\site-packages\numpy\.libs\libopenblas.FB5
AE2TYXYH2IJRDKGDGQ3XBKLKTF43H.gfortran-win_amd64.dll
C:\Users\Francis Yego\anaconda3\lib\site-packages\numpy\.libs\libopenblas64__
v0.3.21-gcc_10_3_0.dll
  warnings.warn("loaded more than 1 DLL from .libs:"
```

Importing data set

In [3]:
```
df=pd.read_excel("Health_sav2b.xlsx")
df.head()
```

Out[3]:

	Gender	cluster_type	Marital status	education	Savings_usage	mobile	NSSF_usage	finhealthscore
**0**	1	1	1	1	1	0	3	2
**1**	1	1	1	1	1	0	3	2
**2**	1	1	1	1	1	0	3	2
**3**	1	1	1	1	1	0	3	2
**4**	1	1	1	1	1	0	3	2

Combining traaining data set for EDA

In [4]:
```python
df.info()
```

```
<class 'pandas.core.frame.DataFrame'>
RangeIndex: 22024 entries, 0 to 22023
Data columns (total 24 columns):
 #   Column                           Non-Null Count  Dtype  
---  ------                           --------------  -----  
 0   Gender                           22024 non-null  int64  
 1   cluster_type                     22024 non-null  int64  
 2   Marital status                   22024 non-null  int64  
 3   education                        22024 non-null  int64  
 4   Savings_usage                    22024 non-null  int64  
 5   mobile                           22024 non-null  int64  
 6   NSSF_usage                       22024 non-null  int64  
 7   finhealthscore                   22024 non-null  int64  
 8   invest                           22024 non-null  int64  
 9   risk_cope                        22024 non-null  int64  
 10  shock                            22024 non-null  int64  
 11  internet                         22024 non-null  int64  
 12  Cryptourrency_bitcoin_Etherium   22024 non-null  int64  
 13  probability_pov_npl              22024 non-null  float64
 14  agegroup2022                     20909 non-null  float64
 15  Percept_gaming                   22024 non-null  int64  
 16  Av_monthly_income                22024 non-null  int64  
 17  Dwelling_tenure                  21954 non-null  float64
 18  defaulted                        22024 non-null  int64  
 19  invest_score                     22024 non-null  int64  
 20  meet_Financial                   19602 non-null  float64
 21  new_wealth                       21954 non-null  float64
 22  formal_banked2022                22024 non-null  int64  
 23  NHIF_health_insurance            22024 non-null  int64  
dtypes: float64(5), int64(19)
memory usage: 4.0 MB
```

In [5]:
```python
df.select_dtypes(include="float").nunique()
```

Out[5]:
```
probability_pov_npl    100
agegroup2022             3
Dwelling_tenure          4
meet_Financial           2
new_wealth               3
dtype: int64
```

In [6]:
```python
df.select_dtypes(include="float").columns
```

Out[6]:
```
Index(['probability_pov_npl', 'agegroup2022', 'Dwelling_tenure',
       'meet_Financial', 'new_wealth'],
      dtype='object')
```

In [7]:
```python
fl=['agegroup2022', 'Dwelling_tenure',
       'meet_Financial', 'new_wealth']
```

In [8]:
```
# df[fl]=df[fl].astype(int)
df.info()
```

```
<class 'pandas.core.frame.DataFrame'>
RangeIndex: 22024 entries, 0 to 22023
Data columns (total 24 columns):
 #   Column                           Non-Null Count  Dtype  
---  ------                           --------------  -----  
 0   Gender                           22024 non-null  int64  
 1   cluster_type                     22024 non-null  int64  
 2   Marital status                   22024 non-null  int64  
 3   education                        22024 non-null  int64  
 4   Savings_usage                    22024 non-null  int64  
 5   mobile                           22024 non-null  int64  
 6   NSSF_usage                       22024 non-null  int64  
 7   finhealthscore                   22024 non-null  int64  
 8   invest                           22024 non-null  int64  
 9   risk_cope                        22024 non-null  int64  
 10  shock                            22024 non-null  int64  
 11  internet                         22024 non-null  int64  
 12  Cryptourrency_bitcoin_Etherium   22024 non-null  int64  
 13  probability_pov_npl              22024 non-null  float64
 14  agegroup2022                     20909 non-null  float64
 15  Percept_gaming                   22024 non-null  int64  
 16  Av_monthly_income                22024 non-null  int64  
 17  Dwelling_tenure                  21954 non-null  float64
 18  defaulted                        22024 non-null  int64  
 19  invest_score                     22024 non-null  int64  
 20  meet_Financial                   19602 non-null  float64
 21  new_wealth                       21954 non-null  float64
 22  formal_banked2022                22024 non-null  int64  
 23  NHIF_health_insurance            22024 non-null  int64  
dtypes: float64(5), int64(19)
memory usage: 4.0 MB
```

In [9]:
```
df['NHIF_health_insurance'].value_counts()
```

Out[9]:
```
0    17367
1     4657
Name: NHIF_health_insurance, dtype: int64
```

Check for missing values if any

In [10]:
```
df.isnull().sum()
```

Out[10]:
```
Gender                              0
cluster_type                        0
Marital status                      0
education                           0
Savings_usage                       0
mobile                              0
NSSF_usage                          0
finhealthscore                      0
invest                              0
risk_cope                           0
shock                               0
internet                            0
Cryptourrency_bitcoin_Etherium      0
probability_pov_npl                 0
agegroup2022                     1115
Percept_gaming                      0
Av_monthly_income                   0
Dwelling_tenure                    70
defaulted                           0
invest_score                        0
meet_Financial                   2422
new_wealth                         70
formal_banked2022                   0
NHIF_health_insurance               0
dtype: int64
```

In [ ]:

In [11]:
```
dfh=df.copy()
# dfh.drop(['Loans default','Transparency',"Uninsured"],1, inplace=True)
```

In [12]:
```
dff=dfh.dropna()
dff.shape
```

Out[12]:
```
(18697, 24)
```

Basic EDA with pandas Profile Report

In [ ]:

In [13]:
```
# import pandas_profiling as pp
# pp.ProfileReport(dff)
```

In [14]:
```
#print(df.apply(LabelEncoder().fit_transform))
objList = dff.select_dtypes(include = "object").columns
print (objList)
```

```
Index([], dtype='object')
```

In [15]:
```
cor= dff.corr()
plt.figure(figsize=(18,18))
sns.heatmap(cor, cbar = True,  square = True, annot=True, fmt= '.2f',annot_kws=
            xticklabels=cor.columns.values,
            yticklabels=cor.columns.values)
```

Out[15]: <AxesSubplot:>

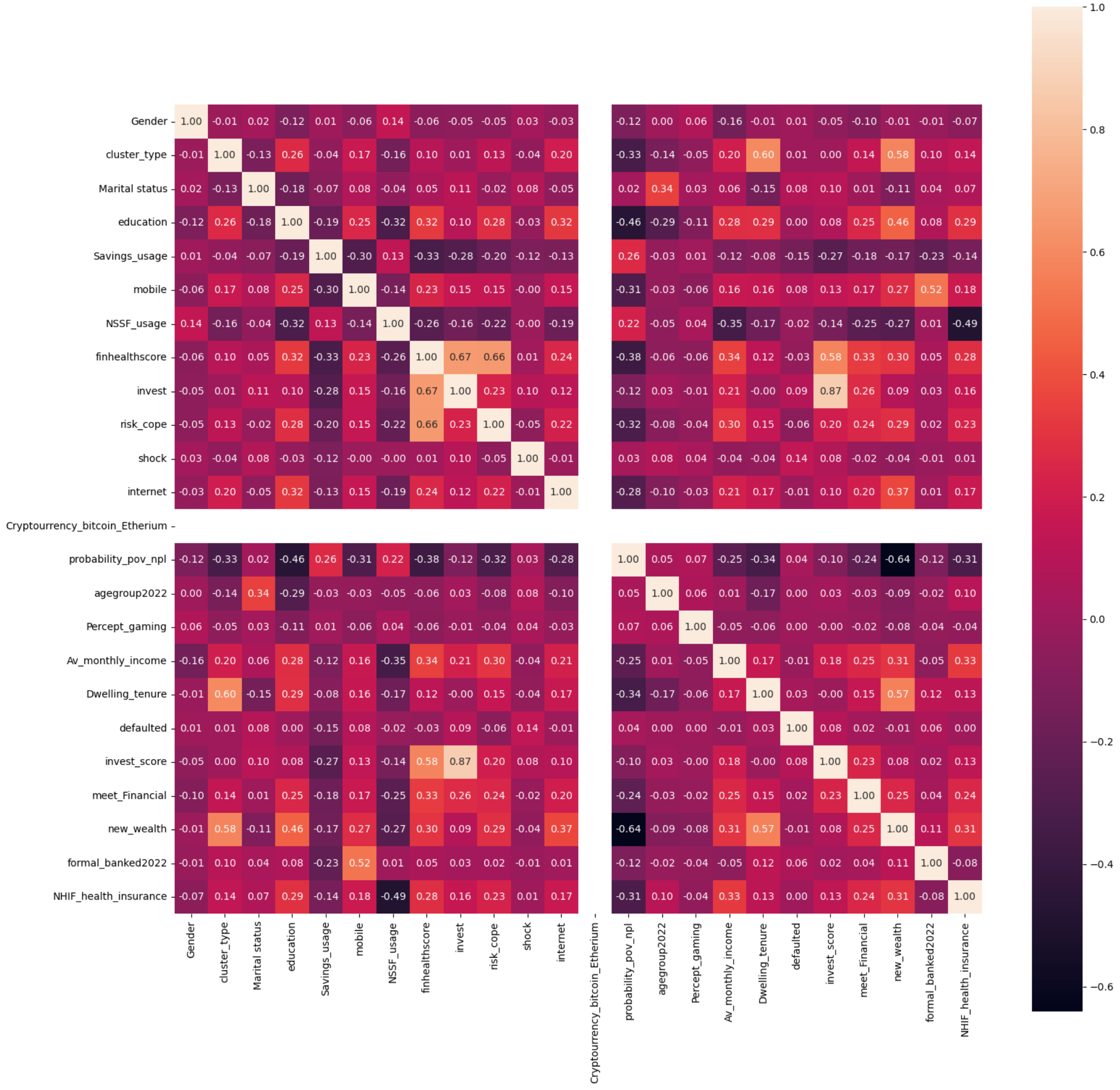

In [16]:
```
plt.figure(figsize=(12,10))
cor = dff.corr()
sns.heatmap(cor, annot=True, cmap=plt.cm.Reds)
plt.show()
```

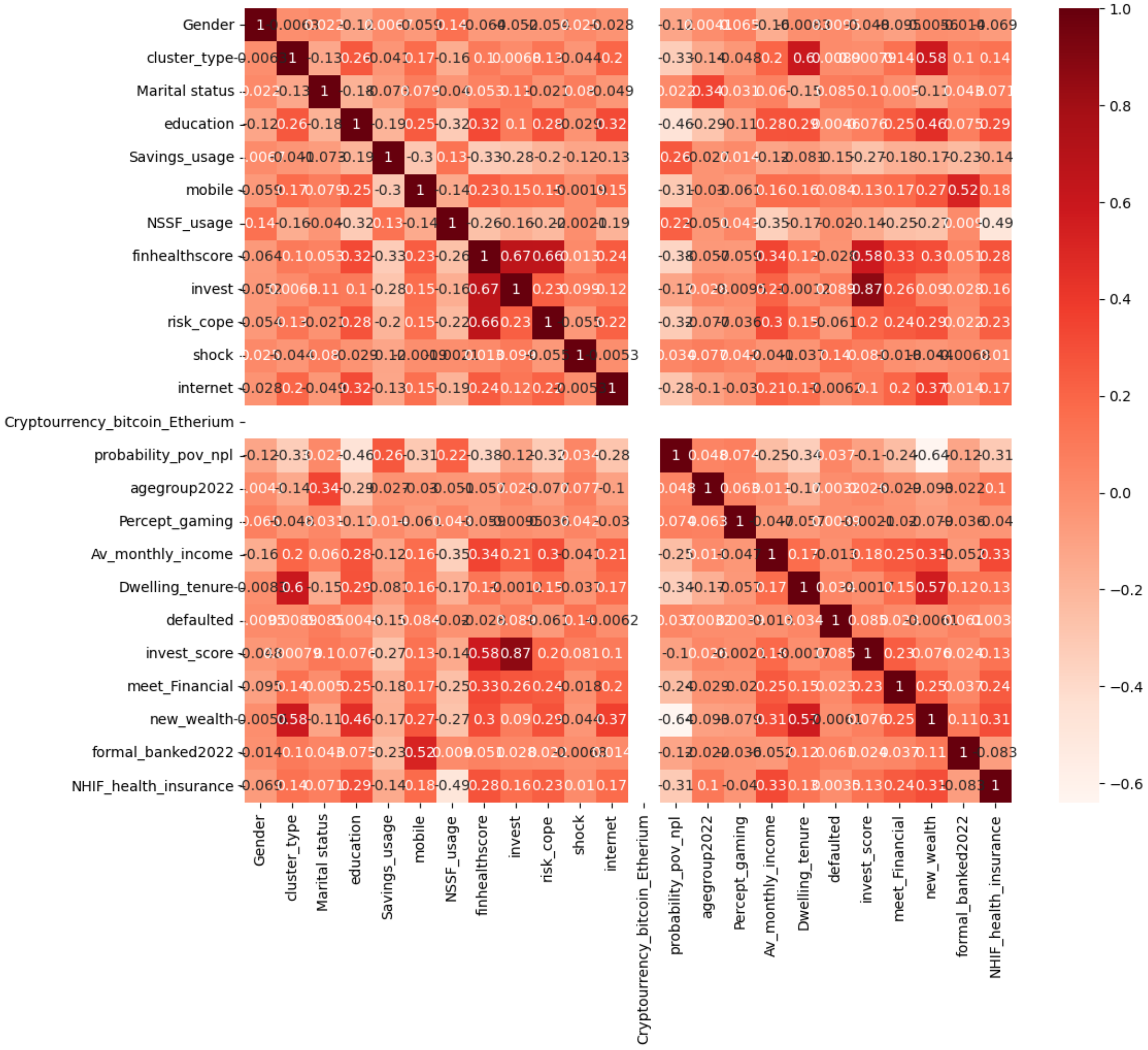

In [17]:
```
#Correlation with output variable
cor_target = abs(cor["NHIF_health_insurance"])
#Selecting highly correlated features
relevant_features = cor_target[cor_target>0.02]
relevant_features.sort_values( ascending=False)
```

Out[17]:
```
NHIF_health_insurance    1.000000
NSSF_usage               0.491912
Av_monthly_income        0.325725
probability_pov_npl      0.308136
new_wealth               0.306677
education                0.289494
finhealthscore           0.280400
meet_Financial           0.244017
risk_cope                0.229085
mobile                   0.176539
internet                 0.169625
invest                   0.158621
Savings_usage            0.142854
cluster_type             0.138246
invest_score             0.130434
Dwelling_tenure          0.127084
agegroup2022             0.102635
formal_banked2022        0.082767
Marital status           0.070612
Gender                   0.069064
Percept_gaming           0.039731
Name: NHIF_health_insurance, dtype: float64
```

In [18]:
```
relevant_features.index
```

Out[18]:
```
Index(['Gender', 'cluster_type', 'Marital status', 'education',
       'Savings_usage', 'mobile', 'NSSF_usage', 'finhealthscore', 'invest',
       'risk_cope', 'internet', 'probability_pov_npl', 'agegroup2022',
       'Percept_gaming', 'Av_monthly_income', 'Dwelling_tenure',
       'invest_score', 'meet_Financial', 'new_wealth', 'formal_banked2022',
       'NHIF_health_insurance'],
      dtype='object')
```

In [19]:
```
# dff=dff[['Selected Respondent Gender', 'Cluster Type (rural/urban)',
#        'Marital status of Respondent', 'Education level of Respondent',
#        'Mobile Ownership', 'finhealthscore', 'Invest', 'Financially healthy',
#        'Risk', 'internet','Probability_Pov_NPL', 'New Age groups', 'New weal
#        'Income group', 'banked', 'Excluded/informal', 'A22. Marital Status',
#        'child_sum', 'healthinsurance']]
```

In [20]:
```
dff['NHIF_health_insurance'].value_counts()
```

Out[20]:
```
0    14571
1     4126
Name: NHIF_health_insurance, dtype: int64
```

In [21]:
```
colors = sns.color_palette()[0]
```

In [22]:
```
#Using Barplot for this distribution

fig,ax=plt.subplots(figsize=(5,4))
term_order = dff['NHIF_health_insurance'].value_counts().index
sns.countplot(x='NHIF_health_insurance', data = dff, color = colors)
plt.title('Distribution of NHIF healthinsurance',fontsize=13);
plt.xlabel('NHIF Health Insurance Uptake', fontsize=11)
ax.set(xticklabels=['Non_Uptake',"Uptake"])
# Adding counts of each term in our data on top of of each bar.
for i in range (dff.NHIF_health_insurance.value_counts().shape[0]):
    count =(round(dff.NHIF_health_insurance.value_counts(normalize=True),2)*10
    ax.text(i, count, '{:0.0f}%'.format(count), ha = 'center', va='baseline')
```

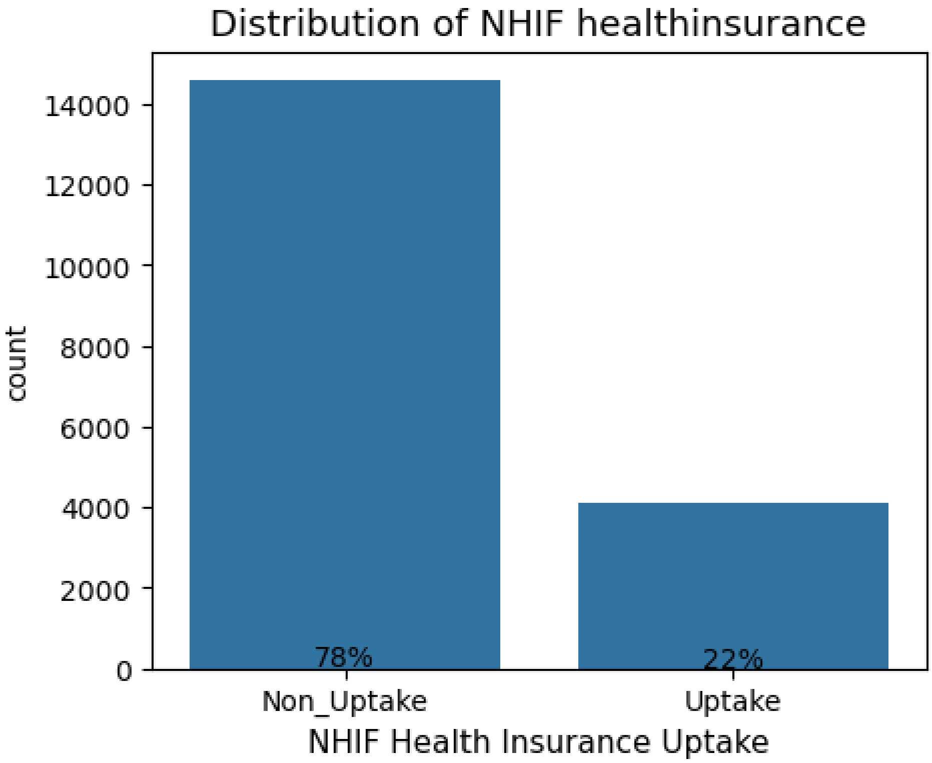

In [23]:
```
colors = ["b", "r"]

sns.countplot('NHIF_health_insurance', data=dff, palette=colors)
plt.title('NHIF healthinsurance Uptake Distributions \n (0: Non healthinsurance
```

Out[23]:
```
Text(0.5, 1.0, 'NHIF healthinsurance Uptake Distributions \n (0: Non healthin
surance Uptake || 1: healthinsurance Uptake)')
```

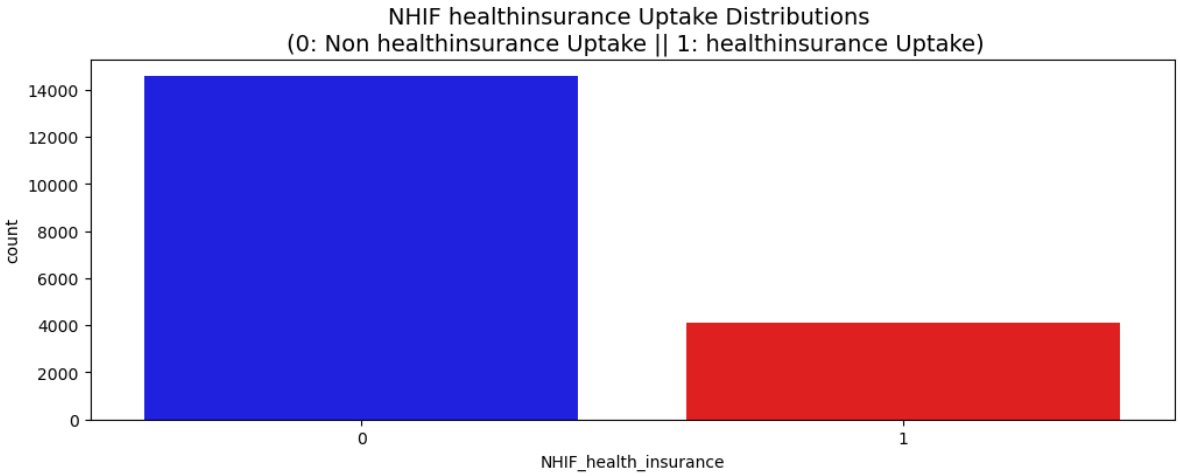

Get the X (data frame consisting depedent varibles)

'Pension usage' dropped because it is the target variable, 'Insurance Usage overall plus nhif and nssf' and 'Mobile Banking Usage' dropped for being redudant while 'Own Land','Major Health problem' and 'Trust' were dropped for having a correlation of less than 0.03 with the target variable.

In [24]:
```
dff.columns
```

Out[24]:
```
Index(['Gender', 'cluster_type', 'Marital status', 'education',
       'Savings_usage', 'mobile', 'NSSF_usage', 'finhealthscore', 'invest',
       'risk_cope', 'shock', 'internet', 'Cryptourrency_bitcoin_Etherium',
       'probability_pov_npl', 'agegroup2022', 'Percept_gaming',
       'Av_monthly_income', 'Dwelling_tenure', 'defaulted', 'invest_score',
       'meet_Financial', 'new_wealth', 'formal_banked2022',
       'NHIF_health_insurance'],
      dtype='object')
```

In [25]:
```
# assuming you have a dataframe called `df` with the original column names

# create a dictionary of new column names
new_names = {
    'cluster_type': 'cluster_type',
    'education': 'education',
    'Savings_usage': 'savings_usage',
    'mobile': 'mobile',
    'NSSF_usage': 'nssf_usage',
    'finhealthscore': 'financial_health_score',
    'invest': 'invest',
    'risk_cope': 'risk_cope',
    'shock': 'shock',
    'internet': 'internet',
    'Cryptourrency_bitcoin_Etherium': 'crypto_currency',
    'probability_pov_npl': 'probability_poverty_index',
    'agegroup2022': 'age_group',
    'Percept_gaming': 'gaming_perception_score',
    'Av_monthly_income': 'average_monthly_income',
    'Dwelling_tenure': 'dwelling_tenure',
    'defaulted': 'defaulted',
    'invest_score': 'investment_score',
    'new_wealth': 'new_wealth_score',
    'formal_banked2022': 'formal_banked',
    'NHIF_health_insurance': 'health_insurance'
}

# rename the columns
dff1 = dff.rename(columns=new_names)
```

In [26]:
```
import pandas as pd

# assuming you have a dataframe called `df` with the original column names

# create a dictionary of new column names
new_names = {
    'gender': 'Gender',
    'cluster_type': 'Cluster Type',
    'marital_status': 'Marital Status',
    'education': 'Education Level',
    'savings_usage': 'Savings Usage',
    'mobile': 'Mobile Ownership',
    'nssf_usage': 'NSSF Enrollment and Usage',
    'financial_health_score': 'Financial Health Score',
    'invest': 'Investment Usage',
    'risk_cope': 'Risk Coping Ability',
    'shock': 'Experienced Shock',
    'internet': 'Internet Access',
    'crypto_currency': 'Cryptocurrency Usage',
    'probability_poverty_index': 'Poverty Vulnerability Score',
    'age_group': 'Age Group',
    'gaming_perception_score': 'Gaming Perception Score',
    'average_monthly_income': 'Average Monthly Income',
    'dwelling_tenure': 'Dwelling Tenure',
    'defaulted': 'Defaulted on Loan Payment',
    'investment_score': 'Investment Score',
    'meet_Financial': 'Meeting Financial Goals Score',
    'new_wealth_score': 'Wealth Quintile Score',
    'formal_banked': 'Formal Bank Account',
    'health_insurance': 'NHIF_health_insurance'
}

# rename the columns
df3 = dff1.rename(columns=new_names)
#Dropping Cryptocurrency Usage due to high multicollinearity
df3.drop(['Cryptocurrency Usage'],axis=1,inplace=True)
```

Correlogram

In [27]:

```
# Calculate Spearman correlation matrix
corr_matrix = df3.corr(method="spearman")

# Plot heatmap
plt.figure(figsize=(9,7))
sns.heatmap(corr_matrix, cmap='gist_stern')
plt.show()
```

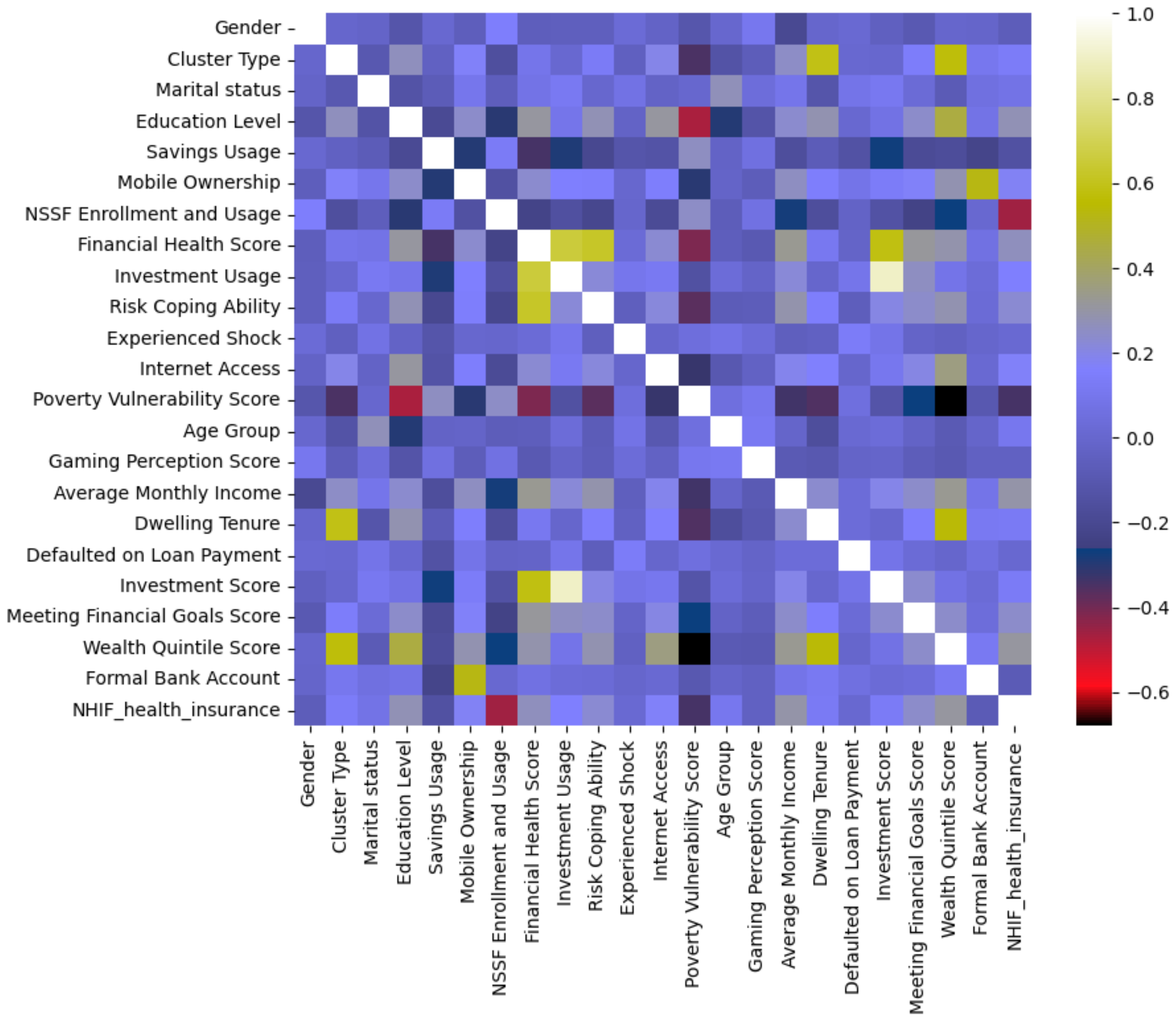

In [28]:

```
X=df3.drop(['NHIF_health_insurance'],axis=1)
```

In [29]:
```
X.columns
```

Out[29]:
```
Index(['Gender', 'Cluster Type', 'Marital status', 'Education Level',
       'Savings Usage', 'Mobile Ownership', 'NSSF Enrollment and Usage',
       'Financial Health Score', 'Investment Usage', 'Risk Coping Ability',
       'Experienced Shock', 'Internet Access', 'Poverty Vulnerability Score',
       'Age Group', 'Gaming Perception Score', 'Average Monthly Income',
       'Dwelling Tenure', 'Defaulted on Loan Payment', 'Investment Score',
       'Meeting Financial Goals Score', 'Wealth Quintile Score',
       'Formal Bank Account'],
      dtype='object')
```

Setting Dummy variables

In [30]:
```
x=pd.get_dummies(X)
y=df3['NHIF_health_insurance']
```

Traditional Logistic Regression

In [31]:
```
import statsmodels.api as sm

# X is your input data (independent variables) and y is your output data (depen
logit_model = sm.Logit(y,x)

# Fit the model
result = logit_model.fit()

# Print summary statistics, including P-values
print(result.summary())
```

```
Optimization terminated successfully.
         Current function value: 0.358598
         Iterations 7
                              Logit Regression Results
==============================================================================
====
Dep. Variable:     NHIF_health_insurance   No. Observations:                  1
8697
Model:                             Logit   Df Residuals:                      1
8675
Method:                              MLE   Df Model:                           
21
Date:                   Sun, 16 Jul 2023   Pseudo R-squ.:                    0.
3205
Time:                           20:49:28   Log-Likelihood:                  -67
04.7
converged:                          True   LL-Null:                         -98
67.6
Covariance Type:               nonrobust   LLR p-value:                        
0.000
==============================================================================
====================
                                  coef    std err          z      P>|z|      
[0.025      0.975]
------------------------------------------------------------------------------
--------------------
Gender                          0.0033      0.045      0.073      0.942      
-0.085       0.092
Cluster Type                   -0.1120      0.060     -1.862      0.063      
-0.230       0.006
Marital status                  0.0679      0.019      3.497      0.000      
0.030       0.106
Education Level                 0.2199      0.028      7.851      0.000      
0.165       0.275
Savings Usage                  -0.0874      0.034     -2.541      0.011      
-0.155      -0.020
Mobile Ownership                1.2317      0.092     13.396      0.000      
1.051       1.412
NSSF Enrollment and Usage      -1.3997      0.035    -40.011      0.000      
-1.468      -1.331
Financial Health Score          0.0690      0.022      3.177      0.001      
0.026       0.112
Investment Usage                0.0544      0.054      1.008      0.314      
-0.051       0.160
Risk Coping Ability             0.0069      0.072      0.096      0.924      
-0.134       0.148
Experienced Shock               0.0933      0.057      1.643      0.100      
-0.018       0.205
Internet Access                -0.1845      0.062     -2.980      0.003      
-0.306      -0.063
Poverty Vulnerability Score    -2.1954      0.151    -14.583      0.000      
-2.490      -1.900
Age Group                       0.6787      0.043     15.943      0.000      
0.595       0.762
Gaming Perception Score        -0.0006      0.001     -0.600      0.548      
-0.003       0.001
Average Monthly Income       2.473e-05   2.54e-06      9.750      0.000      
```

```
1.98e-05    2.97e-05
Dwelling Tenure                   -0.1197      0.031     -3.831      0.000
-0.181      -0.058
Defaulted on Loan Payment          0.0018      0.047      0.039      0.969
-0.090       0.094
Investment Score                  -0.2460      0.090     -2.746      0.006
-0.422      -0.070
Meeting Financial Goals Score      0.3734      0.058      6.390      0.000
0.259       0.488
Wealth Quintile Score              0.3743      0.047      7.957      0.000
0.282       0.467
Formal Bank Account               -1.1151      0.055    -20.397      0.000
-1.222      -1.008
==============================================================================
====================
```

Train test and validation split

We'll generate a train/validation/test three way split.

In [32]:
```python
set_seed=2
train_ratio = 0.7
validation_ratio = 0.15
test_ratio = 0.15
kfold = 5

x_train, x_test, y_train, y_test = train_test_split(x,y, test_size=0.15)
# StratifiedKFold(n_splits=kfold)
# test is now 15% of the initial data set
# validation is now 15% of the initial data set
x_val, x_test, y_val, y_test = train_test_split(x_test, y_test, test_size=test_
```

Models Training

We'll train a binary classifier to predict pension uptake and evaluate the model using some common evaluation metrics.

In [37]:
```python
x_train.shape,x_test.shape,y_train.shape,y_test.shape,
```

Out[37]:
```
((15892, 22), (1403, 22), (15892,), (1403,))
```

In [36]:
```python
clfs = {
'LogisticRegression' : LogisticRegression(random_state=2),
'RandomForest': RandomForestClassifier(random_state=2),
'XGBoost': XGBClassifier(random_state=2)
}
```

In [42]:

```
#code block to test all models in clfs and generate a report
models_report = pd.DataFrame(columns = ['Model', 'kappa_score', 'Recall_score'

for clf, clf_name in zip(clfs.values(), clfs.keys()):
    clf.fit(x_train,y_train)
    y_pred = clf.predict(x_test)
    y_score = clf.score(x_test,y_test)

    #print('Calculating {}'.format(clf_name))
    t = pd.Series({
                     'Model': clf_name,
                     'kappa_score': cohen_kappa_score(y_test, y_pred),
                     'Recall_score': recall_score(y_test, y_pred,average='macro
                     'F1_score': f1_score(y_test, y_pred,average='macro'),
                     #'AUC':metrics.roc_auc_score(y_test, y_pred),
                     'Accuracy': accuracy_score(y_test, y_pred)}

                   )

    models_report = models_report.append(t, ignore_index = True)

models_report
```

```
[08:00:17] WARNING: C:/Users/Administrator/workspace/xgboost-win64_release_1.
5.0/src/learner.cc:1115: Starting in XGBoost 1.3.0, the default evaluation me
tric used with the objective 'binary:logistic' was changed from 'error' to 'l
ogloss'. Explicitly set eval_metric if you'd like to restore the old behavio
r.
```

Out[42]:

	Model	kappa_score	Recall_score	F1_score	Accuracy
**0**	LogisticRegression	0.557407	0.736648	0.776066	0.880257
**1**	RandomForest	0.592209	0.755014	0.794082	0.888097
**2**	XGBoost	0.558416	0.746105	0.777650	0.875980

In [38]:
```
# Function to optimize model using gridsearch
def gridsearch(model, params,x_train, x_test, y_train, y_test, kfold):
    gs = GridSearchCV(model, params, scoring='accuracy', n_jobs=-1, cv=kfold)
    gs.fit(x_train, y_train)
    print ('Best params: ', gs.best_params_)
    print ('Best AUC on Train set: ', gs.best_score_)
    print( 'Best AUC on Test set: ', gs.score(x_test, y_test))

# Function to generate confusion matrix
def confmat(pred, y_test):
    conmat = np.array(confusion_matrix(y_test, pred, labels=[1,0]))
    conf = pd.DataFrame(conmat, index=['Yes', 'No'],
                             columns=['Predicted yes', 'Predicted no'])
    print( conf)

# Function to plot roc curve
def roc(prob, y_test):
    y_score = prob
    fpr = dict()
    tpr = dict()
    roc_auc=dict()
    fpr[1], tpr[1], _ = roc_curve(y_test, y_score)
    roc_auc[1] = auc(fpr[1], tpr[1])
    plt.figure(figsize=[7,7])
    plt.plot(fpr[1], tpr[1], label='Roc curve (area=%0.2f)' %roc_auc[1], linew
    plt.plot([1,0], [1,0], 'k--', linewidth=4)
    plt.xlim([0.0, 1.0])
    plt.ylim([0.0, 1.0])
    plt.xlabel('False Positive rate', fontsize=15)
    plt.ylabel('True Positive rate', fontsize=15)
    plt.title('ROC curve for Credit Default', fontsize=16)
    plt.legend(loc='Lower Right')
    plt.show()

def model(md, x_train, y_train,x_test, y_test):
    md.fit(x_train, y_train)
    pred = md.predict(x_test)
    #prob = md.predict_proba(x_test)[:,1]
    print( ' ' )
    print ('Accuracy on Train set: ', md.score(x_train, y_train))
    print( 'Accuracy on Test set: ', md.score(x_test, y_test))
    print( ' ')
    print(classification_report(y_test, pred))
    print( ' ')
    print('Confusion Matrix',confmat(pred, y_test))

    #roc(prob, y_test)
    return md
```

In [39]:
```
lg=LogisticRegression()
model_lg = model(lg, x_train, y_train,x_test, y_test)
```

```
Accuracy on Train set:  0.8363327460357413
Accuracy on Test set:  0.8317890235210263

              precision    recall  f1-score   support

           0       0.84      0.97      0.90      1096
           1       0.75      0.35      0.47       307

    accuracy                           0.83      1403
   macro avg       0.80      0.66      0.69      1403
weighted avg       0.82      0.83      0.81      1403


     Predicted yes  Predicted no
Yes            106           201
No              35          1061
Confusion Matrix None
```

SMOTE

In [40]:
```
from imblearn.over_sampling import SMOTE
sm=SMOTE(random_state=12,)
xs,ys=sm.fit_resample(x,y)
x_train,x_test,y_train,y_test=train_test_split(xs,ys, test_size=0.15,random_st
```

In [46]:

```
#code block to test all models in clfs and generate a report
models_report = pd.DataFrame(columns = ['Model', 'kappa_score', 'Recall_score'

for clf, clf_name in zip(clfs.values(), clfs.keys()):
    clf.fit(x_train,y_train)
    y_pred = clf.predict(x_test)
    y_score = clf.score(x_test,y_test)

    #print('Calculating {}'.format(clf_name))
    t = pd.Series({
                     'Model': clf_name,
                     'kappa_score': cohen_kappa_score(y_test, y_pred),
                     'Recall_score': recall_score(y_test, y_pred,average='macr
                     'F1_score': f1_score(y_test, y_pred,average='macro'),
                     #'AUC':metrics.roc_auc_score(y_test, y_pred),
                     'Accuracy': accuracy_score(y_test, y_pred)}

                   )

    models_report = models_report.append(t, ignore_index = True)

models_report
```

```
[08:00:23] WARNING: C:/Users/Administrator/workspace/xgboost-win64_release_1.
5.0/src/learner.cc:1115: Starting in XGBoost 1.3.0, the default evaluation me
tric used with the objective 'binary:logistic' was changed from 'error' to 'l
ogloss'. Explicitly set eval_metric if you'd like to restore the old behavio
r.
```

Out[46]:

	Model	kappa_score	Recall_score	F1_score	Accuracy
**0**	LogisticRegression	0.526443	0.762386	0.762544	0.764181
**1**	RandomForest	0.803848	0.901541	0.901899	0.902104
**2**	XGBoost	0.799937	0.899097	0.899834	0.900274

UPSAMPLED

In [41]:

```
from sklearn.utils import resample



# setting up testing and training sets


# concatenate our predictors and dependent variable data back together
X = pd.concat([x, y], axis=1)

# separate minority and majority classes
Non_health_Uptake= X[X['NHIF_health_insurance']==0]
health_Uptake= X[X['NHIF_health_insurance']==1]

# upsample minority
uptake_upsampled = resample(health_Uptake,
                          replace=True, # sample with replacement
                          n_samples=len(Non_health_Uptake), # match number in [illegible]
                          random_state=2) # reproducible results

# combine majority and upsampled minority
upsampled = pd.concat([Non_health_Uptake, uptake_upsampled])

# check new class counts
upsampled['NHIF_health_insurance'].value_counts()
```

Out[41]:

```
0    14571
1    14571
Name: NHIF_health_insurance, dtype: int64
```

In [42]:
```
colors = ["b"]

sns.countplot('NHIF_health_insurance', data=upsampled, palette=colors)
plt.title('NHIF healthinsurance Distributions \n (0: Non healthinsurance Uptake
```

Out[42]:
```
Text(0.5, 1.0, 'NHIF healthinsurance Distributions \n (0: Non healthinsurance
Uptake || 1: healthinsurance Uptake)')
```

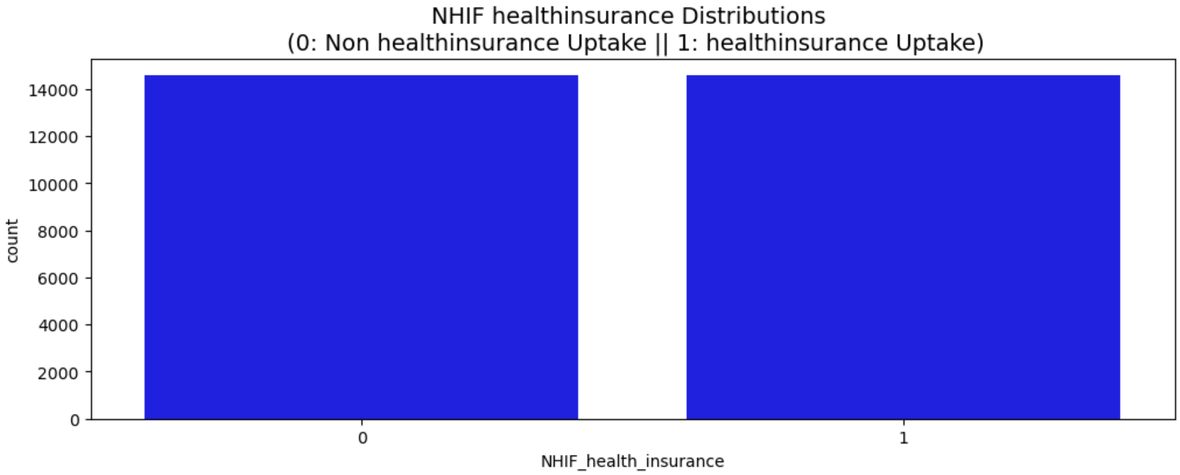

In [43]:
```
# trying logistic regression again with the balanced dataset
y_up = upsampled['NHIF_health_insurance']
x_up= pd.get_dummies(upsampled.drop('NHIF_health_insurance', axis=1))
```

In [49]:
```
train_ratio = 0.7
validation_ratio = 0.15
test_ratio = 0.15
kfold = 5

x_train, x_test, y_train, y_test = train_test_split(x_up,y_up, test_size=0.1,
# StratifiedKFold(n_splits=kfold)
# test is now 15% of the initial data set
# validation is now 15% of the initial data set
x_val, x_test, y_val, y_test = train_test_split(x_test, y_test, test_size=test_
```

In [51]:

```
#code block to test all models in clfs and generate a report
models_report = pd.DataFrame(columns = ['Model', 'kappa_score', 'Recall_score'

for clf, clf_name in zip(clfs.values(), clfs.keys()):
    clf.fit(x_train,y_train)
    y_pred = clf.predict(x_test)
    y_score = clf.score(x_test,y_test)

    #print('Calculating {}'.format(clf_name))
    t = pd.Series({
                     'Model': clf_name,
                     'kappa_score': cohen_kappa_score(y_test, y_pred),
                     'Recall_score': recall_score(y_test, y_pred,average='macr
                     'F1_score': f1_score(y_test, y_pred,average='macro'),
                     #'AUC':metrics.roc_auc_score(y_test, y_pred),
                     'Accuracy': accuracy_score(y_test, y_pred)}

                   )

    models_report = models_report.append(t, ignore_index = True)

models_report
```

```
[08:01:24] WARNING: C:/Users/Administrator/workspace/xgboost-win64_release_1.
5.0/src/learner.cc:1115: Starting in XGBoost 1.3.0, the default evaluation me
tric used with the objective 'binary:logistic' was changed from 'error' to 'l
ogloss'. Explicitly set eval_metric if you'd like to restore the old behavio
r.
```

Out[51]:

	Model	kappa_score	Recall_score	F1_score	Accuracy
**0**	LogisticRegression	0.455647	0.727484	0.727575	0.728395
**1**	RandomForest	0.927310	0.964016	0.963646	0.963649
**2**	XGBoost	0.670492	0.835155	0.835241	0.835391

In [65]:
```
# the models that you want to compare
models = {
    'rf': RandomForestClassifier(random_state=2),
    'xgb': XGBClassifier(random_state=2),
    'lg': LogisticRegression(random_state=2),
    'DT':DecisionTreeClassifier(random_state=2)
}

# the optimisation parameters for each of the above models
params = {
    'rf':{
            "n_estimators"  : [100, 200, 300, 400, 500],
            "criterion": ['gini', 'entropy'],
            "min_samples_leaf": [1,2,3],
            'min_samples_split':[1, 2,3,4]
            },
    'xgb': {
        'learning_rate': [0.05, 0.1, 0.2, 0.3, 0.5],
        'n_estimators': [1000, 1200, 1300, 1400,1500]
        },
    'lg': {
        'solver': ['newton-cg', 'sag', 'liblinear'],
        'penalty': ['l1', 'l2'],
        'fit_intercept':[True, False]
        },
    'DT':{
        "criterion":['gini','entropy']
    }
}
#and you can define:


def fit(x_train, y_train):
        """
        fits the list of models to the training data, thereby obtaining in each
        case an evaluation score after GridSearchCV cross-validation
        """
        for name in models.keys():
            est = models[name]
            est_params = params[name]
            gscv = GridSearchCV(estimator=est, param_grid=est_params, cv=kfold
            gscv.fit(x_train, y_train)
            print("best parameters are: {}".format(gscv.best_estimator_))
```

In [50]:
```
lg=LogisticRegression(random_state=2, solver='newton-cg',penalty='l2',fit_inte
model_lg = model(lg, x_train, y_train,x_test, y_test)
```

```
Accuracy on Train set:  0.7661188851183894
Accuracy on Test set:  0.7860082304526749

              precision    recall  f1-score   support

           0       0.76      0.84      0.80       727
           1       0.82      0.74      0.78       731

    accuracy                           0.79      1458
   macro avg       0.79      0.79      0.79      1458
weighted avg       0.79      0.79      0.79      1458


     Predicted yes  Predicted no
Yes            538           193
No             119           608
Confusion Matrix None
```

In [51]:
```

rf=RandomForestClassifier(n_estimators=100,random_state=2)

model_rf = model(rf, x_train, y_train,x_test, y_test)
```

```
Accuracy on Train set:  0.9999618713539482
Accuracy on Test set:  0.9684499314128944

              precision    recall  f1-score   support

           0       0.98      0.95      0.97       727
           1       0.95      0.98      0.97       731

    accuracy                           0.97      1458
   macro avg       0.97      0.97      0.97      1458
weighted avg       0.97      0.97      0.97      1458


     Predicted yes  Predicted no
Yes            720            11
No              35           692
Confusion Matrix None
```

In [52]:
```
rf=RandomForestClassifier(n_estimators=100,random_state=2)

model_rf = model(rf, x_train, y_train,x_test, y_test)
```

```
Accuracy on Train set:  0.9999618713539482
Accuracy on Test set:  0.9684499314128944

              precision    recall  f1-score   support

           0       0.98      0.95      0.97       727
           1       0.95      0.98      0.97       731

    accuracy                           0.97      1458
   macro avg       0.97      0.97      0.97      1458
weighted avg       0.97      0.97      0.97      1458


     Predicted yes  Predicted no
Yes            720            11
No              35           692
Confusion Matrix None
```

In [48]:
```
# feature selection with the best model from grid search
xgb = XGBClassifier(n_estimators=200,random_state=2)
model_xgb = model(xgb, x_train, y_train,x_test, y_test)
```

```
Accuracy on Train set:  0.9418538147710375
Accuracy on Test set:  0.8854595336076817

              precision    recall  f1-score   support

           0       0.90      0.88      0.89       752
           1       0.87      0.89      0.88       706

    accuracy                           0.89      1458
   macro avg       0.89      0.89      0.89      1458
weighted avg       0.89      0.89      0.89      1458


     Predicted yes  Predicted no
Yes            629            77
No              90           662
Confusion Matrix None
```

In [41]:
```
# feature selection with the best model from grid search
xgb = XGBClassifier(n_estimators=200,random_state=2)
model_xgb = model(xgb, x_train, y_train,x_test, y_test)
```

```
Accuracy on Train set:  0.9418538147710375
Accuracy on Test set:  0.8806584362139918

              precision    recall  f1-score   support

           0       0.89      0.88      0.88       739
           1       0.87      0.89      0.88       719

    accuracy                           0.88      1458
   macro avg       0.88      0.88      0.88      1458
weighted avg       0.88      0.88      0.88      1458


     Predicted yes  Predicted no
Yes            637            82
No              92           647
Confusion Matrix None
```

In [61]:
```
clfs = {
'LogisticRegression' : lg,
'RandomForest': rf,
'XGBoost': xgb
}
#code block to test all models in clfs and generate a report
models_report = pd.DataFrame(columns = ['Model', 'Kappa_score', 'Recall_score'

for clf, clf_name in zip(clfs.values(), clfs.keys()):
    clf.fit(x_train,y_train)
    y_pred = clf.predict(x_test)
    y_score = clf.score(x_test,y_test)

    #print('Calculating {}'.format(clf_name))
    t = pd.Series({
                     'Model': clf_name,
                     'Kappa_score': metrics.cohen_kappa_score(y_test, y_pred),
                     'Recall_score': metrics.recall_score(y_test, y_pred,averag
                     'F1_score': metrics.f1_score(y_test, y_pred,average='macr
#                      'AUC':metrics.roc_auc_score(y_test, y_pred),
                     'Accuracy': metrics.accuracy_score(y_test, y_pred)}

                   )

    models_report = models_report.append(t, ignore_index = True)

models_report
```

Out[61]:

	Model	Kappa_score	Recall_score	F1_score	Accuracy
0	LogisticRegression	0.527642	0.763578	0.763275	0.764060
1	RandomForest	0.928680	0.964450	0.964334	0.964335
2	XGBoost	0.762732	0.881472	0.881342	0.881344

In [56]:
```
clfs = {
'LogisticRegression' : lg,
'RandomForest': rf,
'XGBoost': xgb
}
#code block to test all models in clfs and generate a report
models_report = pd.DataFrame(columns = ['Model', 'Kappa_score', 'Recall_score'

for clf, clf_name in zip(clfs.values(), clfs.keys()):
    clf.fit(x_train,y_train)
    y_pred = clf.predict(x_test)
    y_score = clf.score(x_test,y_test)

    #print('Calculating {}'.format(clf_name))
    t = pd.Series({
                     'Model': clf_name,
                     'Kappa_score': metrics.cohen_kappa_score(y_test, y_pred),
                     'Recall_score': metrics.recall_score(y_test, y_pred,avera
                     'F1_score': metrics.f1_score(y_test, y_pred,average='macr
#                      'AUC':metrics.roc_auc_score(y_test, y_pred),
                     'Accuracy': metrics.accuracy_score(y_test, y_pred)}

                   )

    models_report = models_report.append(t, ignore_index = True)

models_report
```

```
[08:03:55] WARNING: C:/Users/Administrator/workspace/xgboost-win64_release_1.
5.0/src/learner.cc:1115: Starting in XGBoost 1.3.0, the default evaluation me
tric used with the objective 'binary:logistic' was changed from 'error' to 'l
ogloss'. Explicitly set eval_metric if you'd like to restore the old behavio
r.
```

Out[56]:

	Model	Kappa_score	Recall_score	F1_score	Accuracy
**0**	LogisticRegression	0.536357	0.767551	0.767656	0.768861
**1**	RandomForest	0.927310	0.964016	0.963646	0.963649
**2**	XGBoost	0.755797	0.878078	0.877887	0.877915

In [53]:
```
# rf = RandomForestClassifier(n_estimators=100, random_state=2)
# rf.fit(x_train, y_train)  # Assuming X_train and y_train are your training da

# Get predicted probabilities for the positive class (health insurance uptake)
predicted_probabilities = rf.predict_proba(x_test)[:, 1]  # Assuming X_test is
```

In [54]:
```
# Assuming you have trained and fitted the Random Forest model (rf) as before

# Combine the predicted probabilities with the corresponding factors
predictions_with_factors = pd.DataFrame({'Predicted Probability': predicted_pr
                                         'Gender': x_test['Gender'],
                                         'Marital Status': x_test['Marital sta
                                         'Education Level': x_test['Education 
                                         'Mobile Ownership': x_test['Mobile Ow
                                         'Average Monthly Income': x_test['Ave
                                         'Poverty Vulnerability Score': x_test
                                         'Age Group': x_test['Age Group'],
                                         'Wealth Quintile Score': x_test['Weal
                                         'Formal Bank Account': x_test['Formal

# Visualize the distribution of predicted probabilities across the identified 
sns.boxplot(x='Age Group', y='Predicted Probability', data=predictions_with_fa
plt.title('Distribution of Predicted Probabilities by Age Group')
plt.show()

sns.boxplot(x='Wealth Quintile Score', y='Predicted Probability', data=predict
plt.title('Distribution of Predicted Probabilities by Wealth Quintile Score')
plt.show()

sns.boxplot(x='Marital Status', y='Predicted Probability', data=predictions_wi
plt.title('Distribution of Predicted Probabilities by Marital Status')
plt.show()

# You can continue visualizing the remaining factors in a similar manner
```

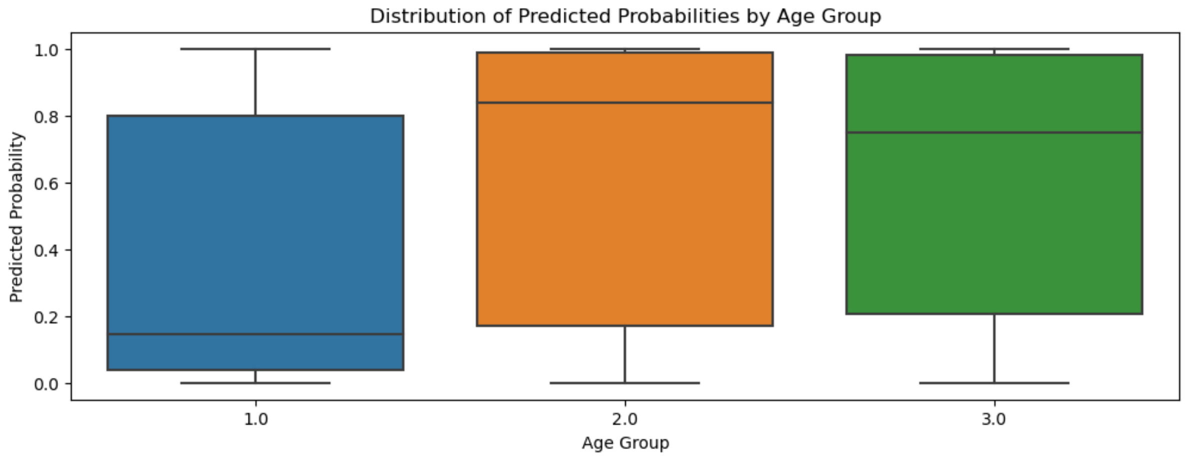

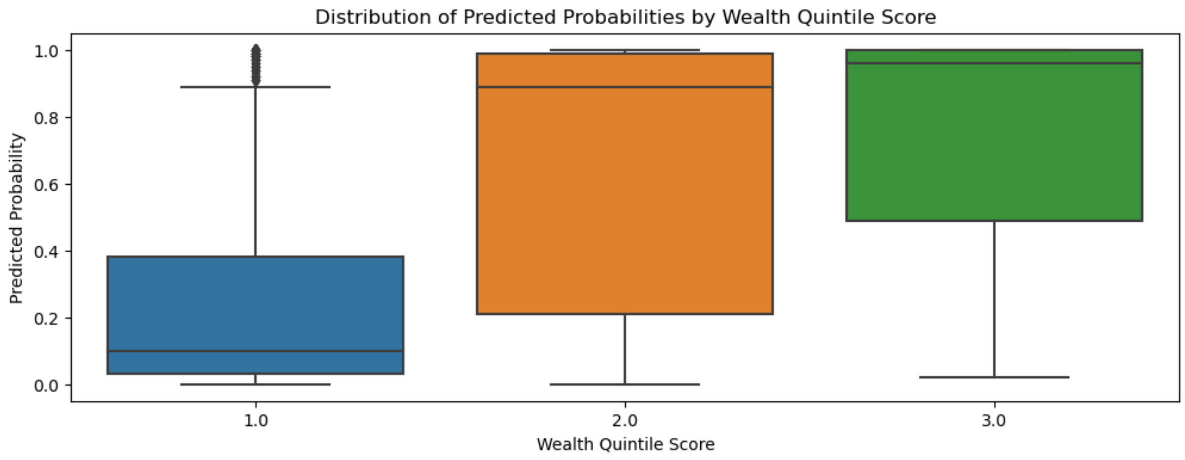

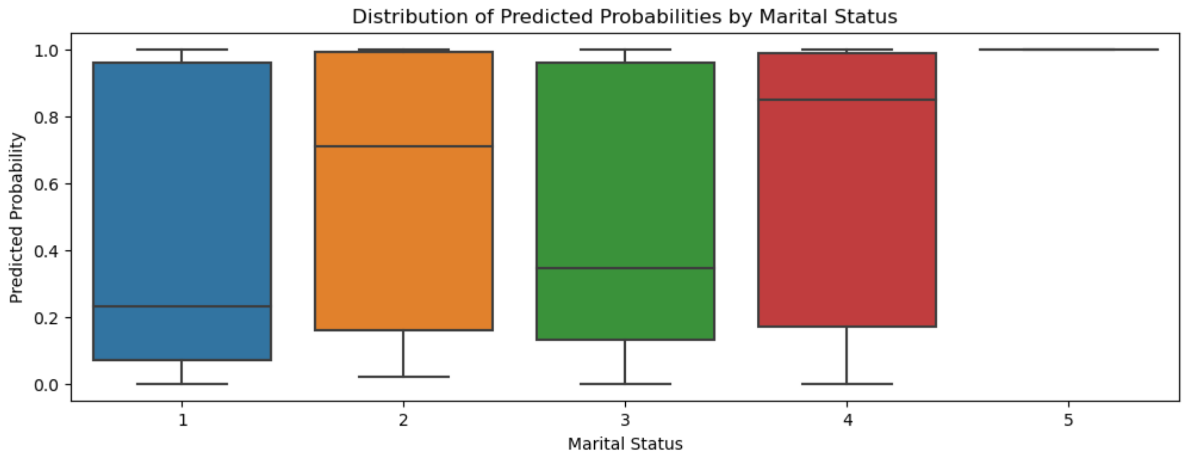

In [57]:
```
x_test.columns
```

Out[57]:
```
Index(['Gender', 'Cluster Type', 'Marital status', 'Education Level',
       'Savings Usage', 'Mobile Ownership', 'NSSF Enrollment and Usage',
       'Financial Health Score', 'Investment Usage', 'Risk Coping Ability',
       'Experienced Shock', 'Internet Access', 'Poverty Vulnerability Score',
       'Age Group', 'Gaming Perception Score', 'Average Monthly Income',
       'Dwelling Tenure', 'Defaulted on Loan Payment', 'Investment Score',
       'Meeting Financial Goals Score', 'Wealth Quintile Score',
       'Formal Bank Account'],
      dtype='object')
```

In [77]:

```
# Combine the predicted probabilities with the corresponding factors
predictions_with_factors = pd.DataFrame({'Predicted Probability': predicted_pr
                                         'Gender': x_test['Gender'],
                                         'Cluster Type': x_test['Cluster Type'
                                         'Marital Status': x_test['Marital sta
                                         'Education Level': x_test['Education 
                                         'Savings Usage': x_test['Savings Usag
                                         'Mobile Ownership': x_test['Mobile Ow
                                         'NSSF Enrollment and Usage': x_test['
                                         'Financial Health Score': x_test['Fin
                                         'Investment Usage': x_test['Investmen
                                         'Risk Coping Ability': x_test['Risk C
                                         'Experienced Shock': x_test['Experien
                                         'Internet Access': x_test['Internet A
                                         'Poverty Vulnerability Score': x_test
                                         'Age Group': x_test['Age Group'],
                                         'Gaming Perception Score': x_test['Ga
                                         'Average Monthly Income': x_test['Ave
                                         'Dwelling Tenure': x_test['Dwelling T
                                         'Defaulted on Loan Payment': x_test['
                                         'Investment Score': x_test['Investmen
                                         'Meeting Financial Goals Score': x_te
                                         'Wealth Quintile Score': x_test['Weal
                                         'Formal Bank Account': x_test['Formal


# Reset the index of the DataFrame
predictions_with_factors = predictions_with_factors.reset_index(drop=True)

# Remove 'Poverty Vulnerability Score' and 'Average Monthly Income' from the l
factors = predictions_with_factors.columns[1:]
factors = factors[~factors.isin(['Poverty Vulnerability Score', 'Average Month

# Define the size of the figure
fig, axes = plt.subplots(nrows=4, ncols=5, figsize=(18, 12))

# Iterate over the factors and create distribution plots
for i, factor in enumerate(factors):
    row = i // 5  # Determine the row index
    col = i % 5  # Determine the column index

    sns.histplot(data=predictions_with_factors, x='Predicted Probability', hue
    axes[row, col].set_title(f'Distribution by {factor}')
    axes[row, col].set_xlabel('Predicted Probability')
    axes[row, col].set_ylabel('Frequency')
plt.suptitle('Distribution of Predicted Probabilities by Factors', fontsize=14
plt.tight_layout()
plt.show()
```

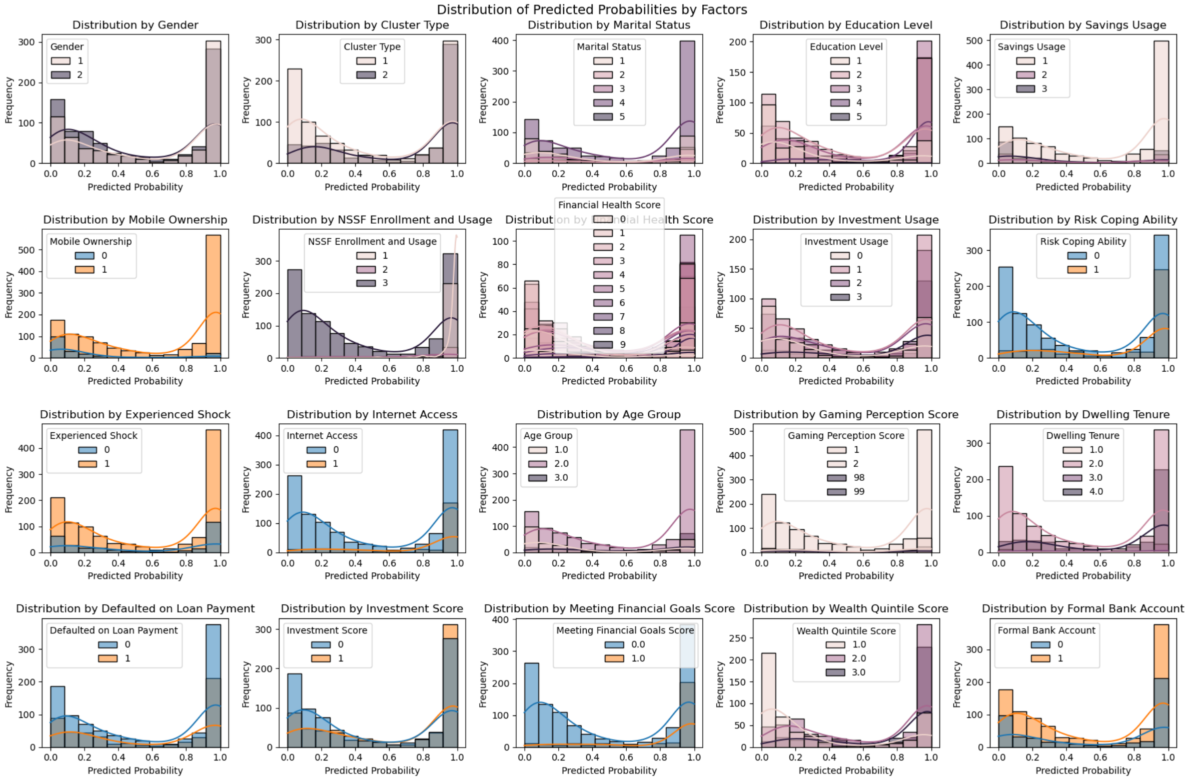
Distribution of Predicted Probabilities by Factors
Distribution by Gender
Distribution by Cluster Type
Distribution by Marital Status
Distribution by Education Level
Distribution by Savings Usage
Distribution by Mobile Ownership
Distribution by NSSF Enrollment and Usage
Distribution by Financial Health Score
Distribution by Investment Usage
Distribution by Risk Coping Ability
Distribution by Experienced Shock
Distribution by Internet Access
Distribution by Age Group
Distribution by Gaming Perception Score
Distribution by Dwelling Tenure
Distribution by Defaulted on Loan Payment
Distribution by Investment Score
Distribution by Meeting Financial Goals Score
Distribution by Wealth Quintile Score
Distribution by Formal Bank Account
Predicted Probability
Frequency

In [74]:
```
# Combine the predicted probabilities with the corresponding factors
predictions_with_factors = pd.DataFrame({'Predicted Probability': predicted_pr
                                         'Gender': x_test['Gender'],
                                         'Cluster Type': x_test['Cluster Type'
                                         'Marital Status': x_test['Marital sta
                                         'Education Level': x_test['Education 
                                         'Savings Usage': x_test['Savings Usag
                                         'Mobile Ownership': x_test['Mobile Ow
                                         'NSSF Enrollment and Usage': x_test['
                                         'Financial Health Score': x_test['Fin
                                         'Investment Usage': x_test['Investmen
                                         'Risk Coping Ability': x_test['Risk C
                                         'Experienced Shock': x_test['Experien
                                         'Internet Access': x_test['Internet A
                                         'Poverty Vulnerability Score': x_test
                                         'Age Group': x_test['Age Group'],
                                         'Gaming Perception Score': x_test['Ga
                                         'Average Monthly Income': x_test['Ave
                                         'Dwelling Tenure': x_test['Dwelling T
                                         'Defaulted on Loan Payment': x_test['
                                         'Investment Score': x_test['Investmen
                                         'Meeting Financial Goals Score': x_te
                                         'Wealth Quintile Score': x_test['Weal
                                         'Formal Bank Account': x_test['Formal
import matplotlib.pyplot as plt
import seaborn as sns

# Create subplots for multiple plots
fig, axes = plt.subplots(4, 6, figsize=(18, 15))

# Flatten the axes array
axes = axes.flatten()

# Iterate over the factors and create violin plots or scatter plots
for i, factor in enumerate(predictions_with_factors.columns[1:]):
    if factor in ['Poverty Vulnerability Score', 'Average Monthly Income']:
        # Scatter plot for continuous variables
        sns.scatterplot(x=factor, y='Predicted Probability', data=predictions_
    else:
        # Violin plot for categorical variables
        sns.violinplot(x=factor, y='Predicted Probability', data=predictions_w

    axes[i].set_title(f'{factor}')
    axes[i].set_xlabel(factor)
    axes[i].set_ylabel('Predicted Probability')

# Adjust the spacing between subplots
plt.tight_layout()

# Display the plot
plt.show()
```

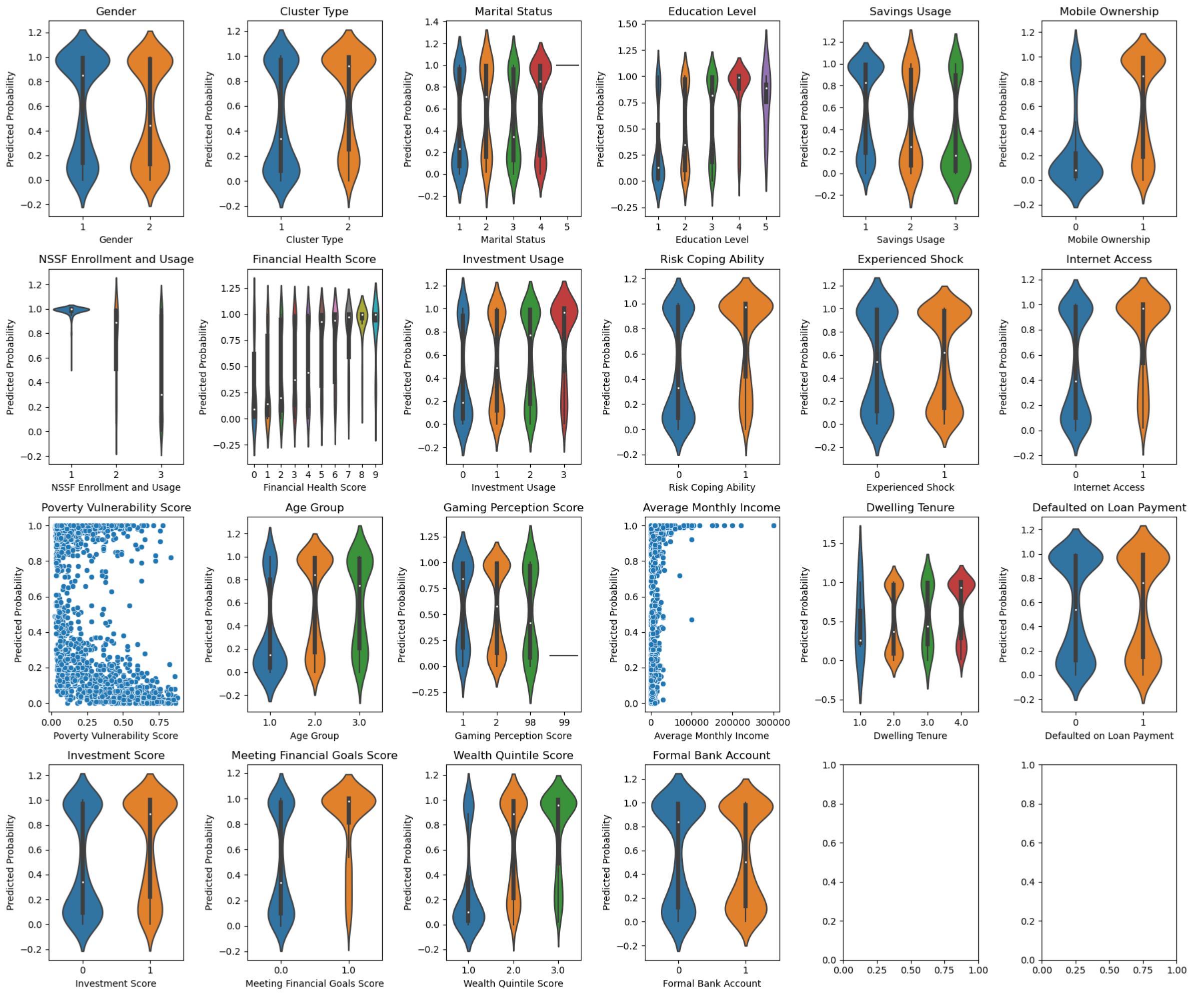

Confusion Matrices

In [57]:
```
from sklearn.metrics import roc_auc_score
```

In [58]:
```
#lg=LogisticRegression(solver='liblinear')
lg.fit(x_train,y_train)
y_pred = lg.predict(x_test)
print(roc_auc_score(y_test,y_pred))
score=cross_val_score(lg,
                        x_train,y_train, cv=10, scoring='roc_auc')
print('Score:',score)
print('AUC',score.mean(),'+',score.std())
plot_confusion_matrix(confusion_matrix(y_test, y_pred))
plt.title('Confusion Matrix (Logistic Regression)')
plt.figure(figsize=(2,2));
```

```
0.7675512175283412
Score: [0.84975227 0.85953468 0.85959515 0.86199395 0.84370589 0.84193091
 0.85223311 0.85883499 0.84959027 0.83753299]
AUC 0.851470420584525 + 0.008053793281369443
```

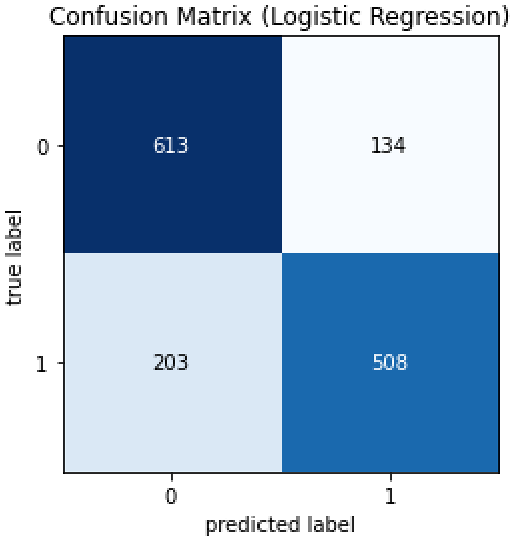


```
<Figure size 144x144 with 0 Axes>
```

In [59]:
```
#lg=LogisticRegression(solver='liblinear')
rf.fit(x_train,y_train)
y_pred = rf.predict(x_test)
print(roc_auc_score(y_test,y_pred))
score=cross_val_score(rf,
                        x_train,y_train, cv=10, scoring='roc_auc')
print('Score:',score)
print('AUC',score.mean(),'+',score.std())
plot_confusion_matrix(confusion_matrix(y_test, y_pred))
plt.title('Confusion Matrix (Random Forest)')
plt.figure(figsize=(2,2));
```

```
0.9640164031654043
Score: [0.99066642 0.99048968 0.99151437 0.98982748 0.98860391 0.98690538
 0.99070825 0.99101682 0.99116577 0.98669498]
AUC 0.9897593061051163 + 0.0016664656586051433
```

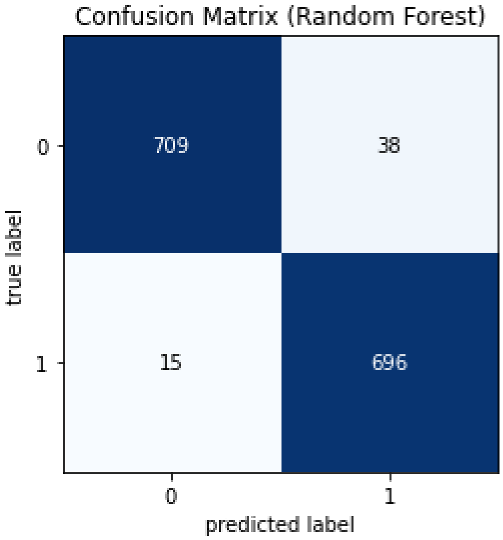


```
<Figure size 144x144 with 0 Axes>
```

In [60]:
```
import warnings
warnings.filterwarnings('ignore')
```

In [61]:
```
xgb.fit(x_train,y_train)
y_pred = xgb.predict(x_test)
print(roc_auc_score(y_test,y_pred))
score=cross_val_score(xgb,
                        x_train,y_train, cv=10, scoring='roc_auc')
print('Score:',score)
print('AUC',score.mean(),'+',score.std())
plot_confusion_matrix(confusion_matrix(y_test, y_pred))
plt.title('Confusion Matrix (XGBoost)')
plt.figure(figsize=(2,2));
```

```
[08:05:22] WARNING: C:/Users/Administrator/workspace/xgboost-win64_release_1.
5.0/src/learner.cc:1115: Starting in XGBoost 1.3.0, the default evaluation me
tric used with the objective 'binary:logistic' was changed from 'error' to 'l
ogloss'. Explicitly set eval_metric if you'd like to restore the old behavio
r.
0.8780777116906444
[08:05:29] WARNING: C:/Users/Administrator/workspace/xgboost-win64_release_1.
5.0/src/learner.cc:1115: Starting in XGBoost 1.3.0, the default evaluation me
tric used with the objective 'binary:logistic' was changed from 'error' to 'l
ogloss'. Explicitly set eval_metric if you'd like to restore the old behavio
r.
[08:05:35] WARNING: C:/Users/Administrator/workspace/xgboost-win64_release_1.
5.0/src/learner.cc:1115: Starting in XGBoost 1.3.0, the default evaluation me
tric used with the objective 'binary:logistic' was changed from 'error' to 'l
ogloss'. Explicitly set eval_metric if you'd like to restore the old behavio
r.
[08:05:40] WARNING: C:/Users/Administrator/workspace/xgboost-win64_release_1.
5.0/src/learner.cc:1115: Starting in XGBoost 1.3.0, the default evaluation me
tric used with the objective 'binary:logistic' was changed from 'error' to 'l
ogloss'. Explicitly set eval_metric if you'd like to restore the old behavio
r.
[08:05:46] WARNING: C:/Users/Administrator/workspace/xgboost-win64_release_1.
5.0/src/learner.cc:1115: Starting in XGBoost 1.3.0, the default evaluation me
tric used with the objective 'binary:logistic' was changed from 'error' to 'l
ogloss'. Explicitly set eval_metric if you'd like to restore the old behavio
r.
[08:05:51] WARNING: C:/Users/Administrator/workspace/xgboost-win64_release_1.
5.0/src/learner.cc:1115: Starting in XGBoost 1.3.0, the default evaluation me
tric used with the objective 'binary:logistic' was changed from 'error' to 'l
ogloss'. Explicitly set eval_metric if you'd like to restore the old behavio
r.
[08:05:56] WARNING: C:/Users/Administrator/workspace/xgboost-win64_release_1.
5.0/src/learner.cc:1115: Starting in XGBoost 1.3.0, the default evaluation me
tric used with the objective 'binary:logistic' was changed from 'error' to 'l
ogloss'. Explicitly set eval_metric if you'd like to restore the old behavio
r.
[08:06:00] WARNING: C:/Users/Administrator/workspace/xgboost-win64_release_1.
5.0/src/learner.cc:1115: Starting in XGBoost 1.3.0, the default evaluation me
tric used with the objective 'binary:logistic' was changed from 'error' to 'l
ogloss'. Explicitly set eval_metric if you'd like to restore the old behavio
r.
[08:06:06] WARNING: C:/Users/Administrator/workspace/xgboost-win64_release_1.
5.0/src/learner.cc:1115: Starting in XGBoost 1.3.0, the default evaluation me
tric used with the objective 'binary:logistic' was changed from 'error' to 'l
ogloss'. Explicitly set eval_metric if you'd like to restore the old behavio
r.
[08:06:10] WARNING: C:/Users/Administrator/workspace/xgboost-win64_release_1.
5.0/src/learner.cc:1115: Starting in XGBoost 1.3.0, the default evaluation me
tric used with the objective 'binary:logistic' was changed from 'error' to 'l
ogloss'. Explicitly set eval_metric if you'd like to restore the old behavio
r.
[08:06:14] WARNING: C:/Users/Administrator/workspace/xgboost-win64_release_1.
5.0/src/learner.cc:1115: Starting in XGBoost 1.3.0, the default evaluation me
tric used with the objective 'binary:logistic' was changed from 'error' to 'l
ogloss'. Explicitly set eval_metric if you'd like to restore the old behavio
r.
Score: [0.9504618  0.94848159 0.95097923 0.94719731 0.94402333 0.94161347
```

```
 0.94561052 0.95277161 0.95227473 0.9431202 ]
AUC 0.9476533772343879 + 0.0037653205668975094
```

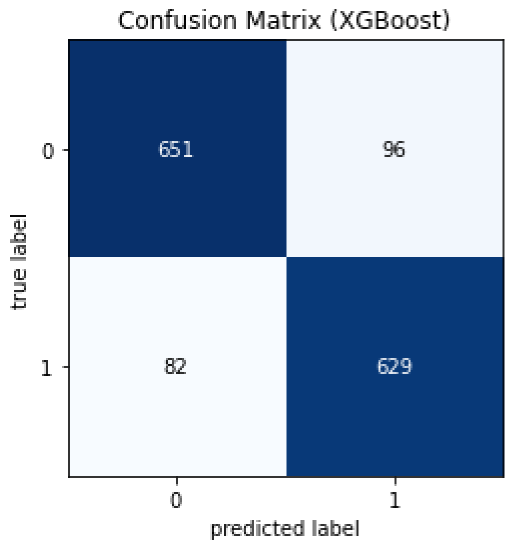


```
<Figure size 144x144 with 0 Axes>
```

Area Under Receiver Operating Characteristic Curve (AUC)

In [62]:
```
probas_train = lg.predict_proba(x_train)
fpr_t, tpr_t, thresholds_t = roc_curve(y_train, probas_train[:, 1])
trai=auc(fpr_t, tpr_t)
# Testing Set
probas_ = lg.predict_proba(x_test)
fpr_lg, tpr_lg, thresholds = roc_curve(y_test, probas_[:, 1])
roc_auc_lg = auc(fpr_lg, tpr_lg)

train, = plt.plot(fpr_t, tpr_t, label = 'Train=' +str(round(trai,4)))
test, = plt.plot(fpr_lg, tpr_lg, label = 'Test=' +str(round(roc_auc_lg,4)))
plt.xlabel('False Positive Rate')
plt.ylabel('True Positive Rate')
plt.legend()
plt.show()

print('Area Under the Curve (AUC): ', roc_auc_lg)
```

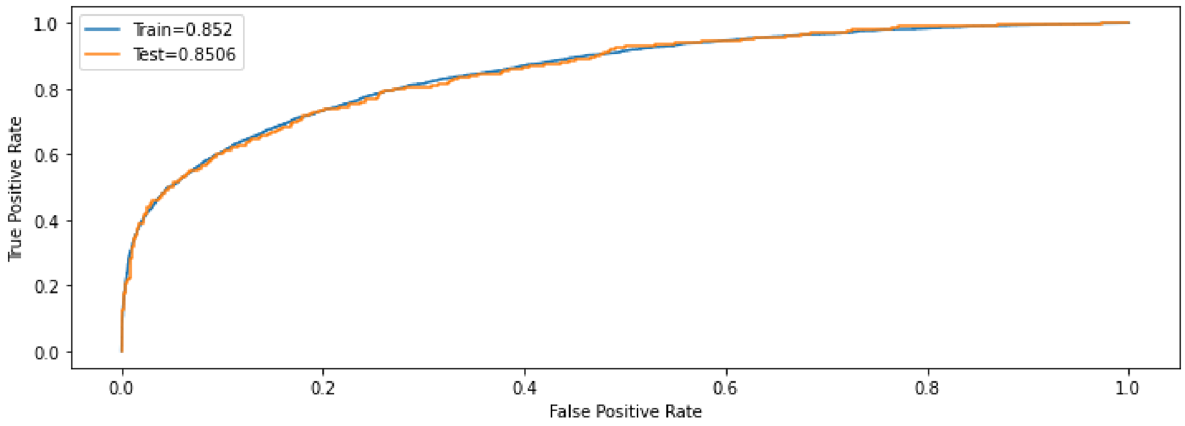


```
Area Under the Curve (AUC):  0.850588476738647
```

In [63]:
```
probas_train = rf.predict_proba(x_train)
fpr_t, tpr_t, thresholds_t = roc_curve(y_train, probas_train[:, 1])
trai=auc(fpr_t, tpr_t)
# Testing Set
probas_ = rf.predict_proba(x_test)
fpr_rf, tpr_rf, thresholds = roc_curve(y_test, probas_[:, 1])
roc_auc_rf= auc(fpr_rf, tpr_rf)

train, = plt.plot(fpr_t, tpr_t, label = 'Train=' +str(round(trai,4)))
test, = plt.plot(fpr_rf, tpr_rf, label = 'Test=' +str(round(roc_auc_rf,4)))
plt.xlabel('False Positive Rate')
plt.ylabel('True Positive Rate')
plt.legend()
plt.show()

print('Area Under the Curve (AUC): ', roc_auc_rf)
```

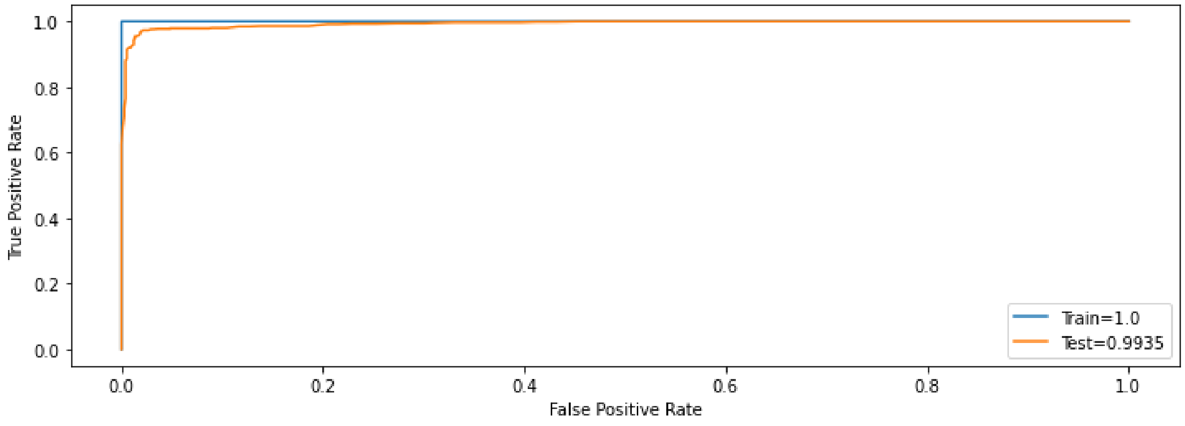


```
Area Under the Curve (AUC):  0.9934769551718359
```

In [64]:
```
probas_train = xgb.predict_proba(x_train)
fpr_t, tpr_t, thresholds_t = roc_curve(y_train, probas_train[:, 1])

# Testing Set
probas_ = xgb.predict_proba(x_test)
fpr_XGB, tpr_XGB, thresholds = roc_curve(y_test, probas_[:, 1])
roc_auc_XGB = auc(fpr_XGB, tpr_XGB)

train, = plt.plot(fpr_t, tpr_t, label = 'Train')
test, = plt.plot(fpr_XGB, tpr_XGB, label = 'Test')
plt.xlabel('False Positive Rate')
plt.ylabel('True Positive Rate')
plt.legend()
plt.show()

print('Area Under the Curve for XGB: ', roc_auc_XGB)
```

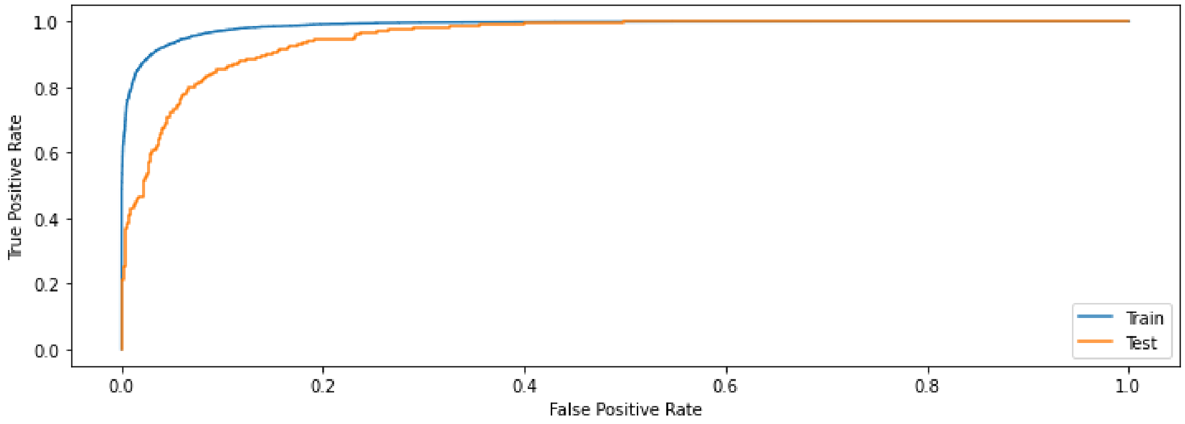


```
Area Under the Curve for XGB:  0.9515436335120132
```

Since AUC is 0.9912, it means there is 99.12% chance that model will be able to distinguish between positive class and negative class.

In [66]:
```
plt.figure(figsize=(7,7))
test, = plt.plot(fpr_lg, tpr_lg, label = 'logistic regression=' +str(round(roc_
test, = plt.plot(fpr_rf, tpr_rf, label ='Random forest=' +str(round(roc_auc_rf
test, = plt.plot(fpr_XGB, tpr_XGB, label = 'XGBoost=' +str(round(roc_auc_XGB,4
plt.plot([0,1],[0,1],'k--')
plt.xlabel('False Positive Rate')
plt.ylabel('True Positive Rate')
plt.title('Area Under Receiver Operating Characteristic Curve (AUC)',fontsize=
plt.annotate('At AUC of 0.5', xy=(0.5, 0.5), xytext=(0.6, 0.3),
                arrowprops=dict(facecolor='b', shrink=0.05),
                )
plt.legend(fontsize=15)
plt.grid()
plt.show()
```

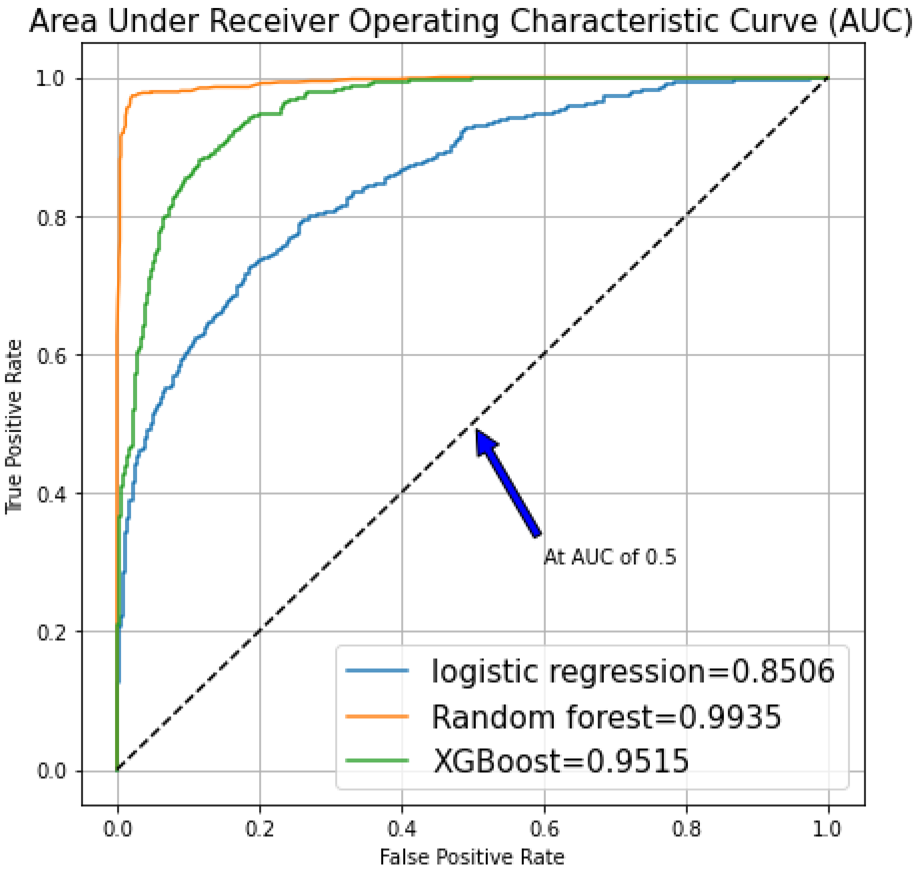

Feature Importance

In [62]:
```
from yellowbrick.model_selection import FeatureImportances
from yellowbrick.exceptions import YellowbrickValueError
plt.figure(figsize=(6,5))

model = lg
viz = FeatureImportances(model,colors=colors)
viz.fit(x, y)
viz.show()
```

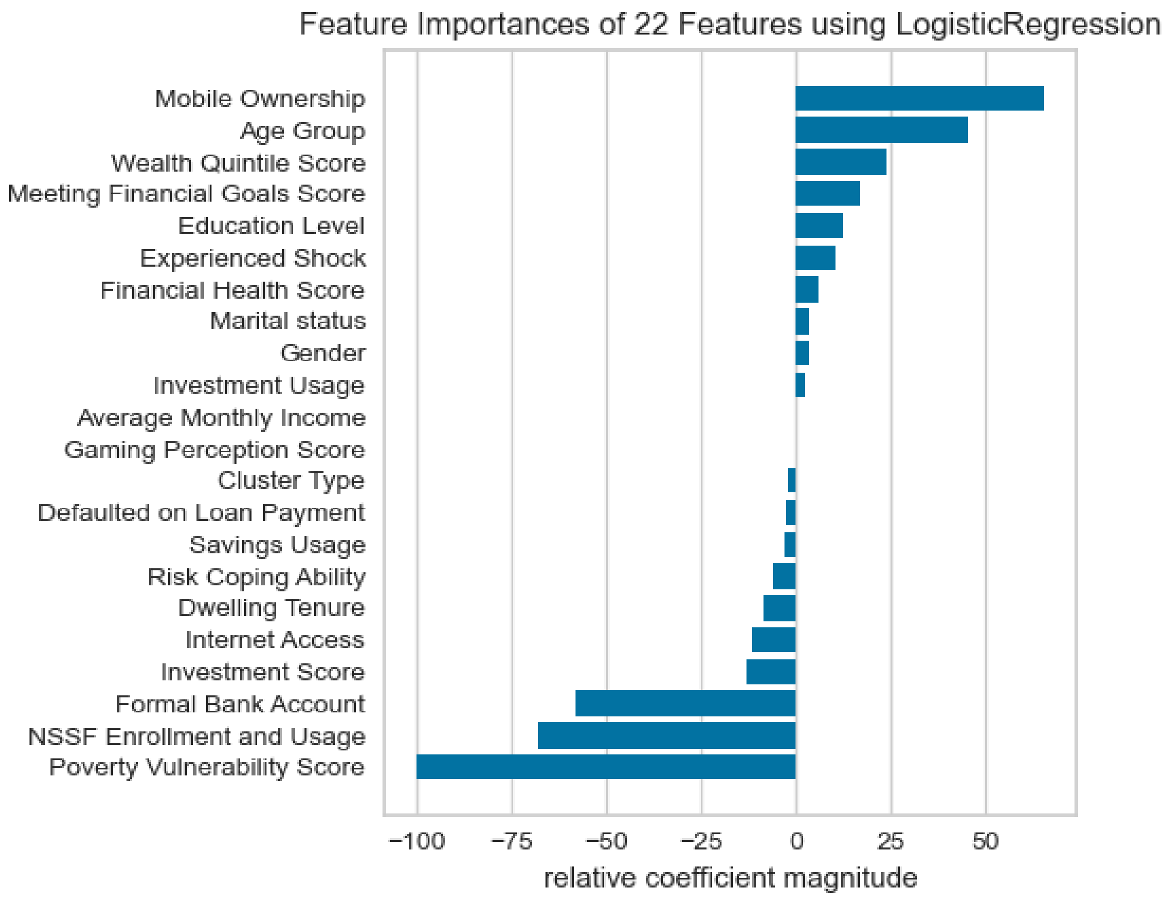

Out[62]:
```
<AxesSubplot:title={'center':'Feature Importances of 22 Features using Logist
icRegression'}, xlabel='relative coefficient magnitude'>
```

In [70]:
```
# Assuming your logistic regression model is named 'lg'
import pandas as pd

# Create a pandas DataFrame with feature names and coefficients
feature_importances = pd.DataFrame(
    {'feature': x.columns, 'importance': lg.coef_[0]}
)

# Sort the DataFrame by feature importance in descending order
feature_importances = feature_importances.sort_values('importance', ascending=

# Print the sorted DataFrame
print(feature_importances)
```

```
                          feature  importance
5                Mobile Ownership    1.217849
13                      Age Group    0.674696
20          Wealth Quintile Score    0.379093
19  Meeting Financial Goals Score    0.372929
3                 Education Level    0.220122
10              Experienced Shock    0.091607
7          Financial Health Score    0.069730
2                  Marital status    0.067968
8                Investment Usage    0.052515
9              Risk Coping Ability    0.007158
0                          Gender    0.004150
17       Defaulted on Loan Payment    0.000992
15          Average Monthly Income    0.000025
14         Gaming Perception Score   -0.000617
4                   Savings Usage   -0.089950
1                    Cluster Type   -0.112804
16                 Dwelling Tenure   -0.120531
11                 Internet Access   -0.182760
18                Investment Score   -0.243514
21             Formal Bank Account   -1.109824
6         NSSF Enrollment and Usage   -1.398829
12     Poverty Vulnerability Score   -2.148061
```

In [71]:
```
# Import necessary libraries
import pandas as pd
from sklearn.linear_model import LogisticRegression

# Fit the logistic regression model
lg.fit(x, y)

# Create a pandas DataFrame with feature importances
feature_importances = pd.DataFrame(
    {'feature': x.columns, 'importance': abs(lg.coef_[0])}
)

# Sort the DataFrame by feature importance in descending order
feature_importances = feature_importances.sort_values('importance', ascending=

# Print the sorted DataFrame
print(feature_importances)
```

```
                          feature  importance
12    Poverty Vulnerability Score    2.148061
6        NSSF Enrollment and Usage    1.398829
5                 Mobile Ownership    1.217849
21             Formal Bank Account    1.109824
13                        Age Group    0.674696
20            Wealth Quintile Score    0.379093
19  Meeting Financial Goals Score    0.372929
18                 Investment Score    0.243514
3                   Education Level    0.220122
11                  Internet Access    0.182760
16                  Dwelling Tenure    0.120531
1                      Cluster Type    0.112804
10               Experienced Shock    0.091607
4                     Savings Usage    0.089950
7           Financial Health Score    0.069730
2                   Marital status    0.067968
8                 Investment Usage    0.052515
9              Risk Coping Ability    0.007158
0                           Gender    0.004150
17        Defaulted on Loan Payment    0.000992
14          Gaming Perception Score    0.000617
15           Average Monthly Income    0.000025
```

In [68]:
```
x.columns = [x.lower() for x in x.columns]
feature_importance = rf.feature_importances_
# make importances relative to max importance
#feature_importance = 100.0 * (feature_importance / feature_importance.max())
sorted_idx = np.argsort(feature_importance)
pos = np.arange(sorted_idx.shape[0]) + .5
# plt.subplot(1, 2, 2)
color=['blue']
plt.figure(figsize=(6, 5))
plt.barh(pos, feature_importance[sorted_idx], align='center',color=colors)
plt.yticks(pos, x.keys()[sorted_idx])
plt.xlabel('Relative Importance')
plt.title('Variable Importance for Random Forest',fontsize=15)
plt.show()
```

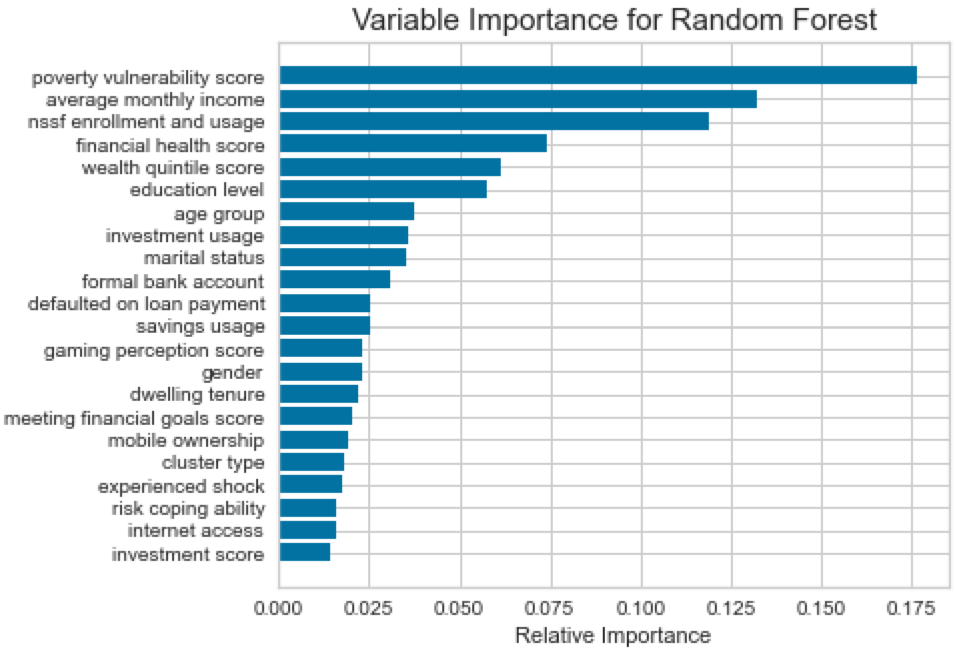

In [72]:
```
import pandas as pd

# Assuming your random forest model is named 'rf'

# Create a Pandas DataFrame with feature importances
feature_importances = pd.DataFrame(
    {'feature': x.columns, 'importance': rf.feature_importances_}
)

# Sort the DataFrame by feature importance in descending order
feature_importances = feature_importances.sort_values('importance', ascending=

# Print the sorted DataFrame
print(feature_importances)
```

```
                          feature  importance
12    Poverty Vulnerability Score    0.176523
15         Average Monthly Income    0.131963
6        NSSF Enrollment and Usage    0.118883
7          Financial Health Score    0.073895
20          Wealth Quintile Score    0.061528
3                 Education Level    0.057322
13                      Age Group    0.037702
8                Investment Usage    0.036145
2                  Marital status    0.035091
21            Formal Bank Account    0.030935
17      Defaulted on Loan Payment    0.025254
4                   Savings Usage    0.025164
14         Gaming Perception Score    0.023347
0                          Gender    0.023080
16                Dwelling Tenure    0.022139
19  Meeting Financial Goals Score    0.020529
5                Mobile Ownership    0.019076
1                    Cluster Type    0.018114
10              Experienced Shock    0.017632
9              Risk Coping Ability    0.015960
11                Internet Access    0.015684
18               Investment Score    0.014037
```

In [69]:
```
x.columns = [x.lower() for x in x.columns]
feature_importance = xgb.feature_importances_
# make importances relative to max importance
#feature_importance = 100.0 * (feature_importance / feature_importance.max())
sorted_idx = np.argsort(feature_importance)
pos = np.arange(sorted_idx.shape[0]) + .5
# plt.subplot(1, 2, 2)
color=['o','b','g','y','k','#66b3ff','#99ff99','#ff9999']
plt.figure(figsize=(6, 5))
plt.barh(pos, feature_importance[sorted_idx], align='center',color=colors)
plt.yticks(pos, x.keys()[sorted_idx])
plt.xlabel('Relative Importance')
plt.title('Variable Importance for XGBoost',fontsize=15)
plt.show()
```

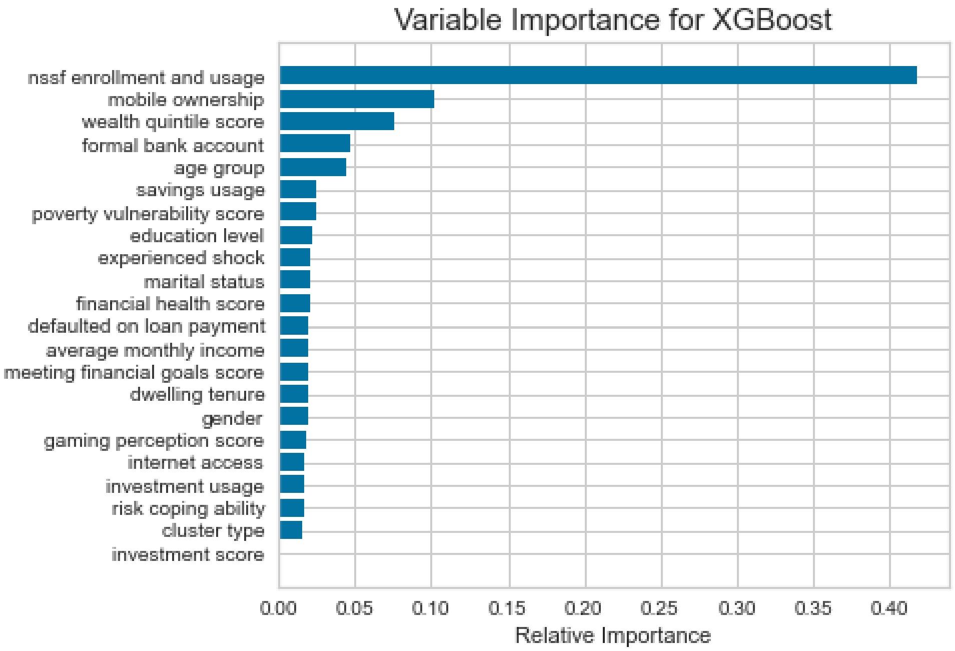

In [73]:
```
import pandas as pd

# Assuming your random forest model is named 'rf'

# Create a Pandas DataFrame with feature importances
feature_importances = pd.DataFrame(
    {'feature': x.columns, 'importance': xgb.feature_importances_}
)

# Sort the DataFrame by feature importance in descending order
feature_importances = feature_importances.sort_values('importance', ascending=

# Print the sorted DataFrame
print(feature_importances)
```

```
                          feature  importance
6        NSSF Enrollment and Usage    0.418172
5                 Mobile Ownership    0.101540
20           Wealth Quintile Score    0.075644
21             Formal Bank Account    0.047393
13                       Age Group    0.044963
4                    Savings Usage    0.024746
12    Poverty Vulnerability Score    0.024463
3                  Education Level    0.022287
10              Experienced Shock    0.021113
2                   Marital status    0.020398
7           Financial Health Score    0.020364
17        Defaulted on Loan Payment   0.019948
15           Average Monthly Income   0.019464
19  Meeting Financial Goals Score    0.019196
16                  Dwelling Tenure   0.018787
0                            Gender   0.018786
14          Gaming Perception Score   0.017847
11                  Internet Access   0.016653
8                 Investment Usage    0.016266
9               Risk Coping Ability   0.016154
1                     Cluster Type    0.015815
18                Investment Score    0.000000
```

In [71]:
```
model.fit(x, y)
# get importance
importance6 = model.coef_[0]
# summarize feature importance
for i,v in enumerate(importance6):
    print('Feature: %0d, Score: %.5f' % (i,v))
```

```
Feature: 0, Score: 0.00415
Feature: 1, Score: -0.11280
Feature: 2, Score: 0.06797
Feature: 3, Score: 0.22012
Feature: 4, Score: -0.08995
Feature: 5, Score: 1.21785
Feature: 6, Score: -1.39883
Feature: 7, Score: 0.06973
Feature: 8, Score: 0.05251
Feature: 9, Score: 0.00716
Feature: 10, Score: 0.09161
Feature: 11, Score: -0.18276
Feature: 12, Score: -2.14806
Feature: 13, Score: 0.67470
Feature: 14, Score: -0.00062
Feature: 15, Score: 0.00002
Feature: 16, Score: -0.12053
Feature: 17, Score: 0.00099
Feature: 18, Score: -0.24351
[illegible]
```

In [72]:
```
# model.fit(x / np.std(x, 0), y)
# # get importance
# importance6 = model.coef_[0]
# importances6 = pd.DataFrame({'feature':x.columns,'importance':np.round(impor
# importances6 = importances6.sort_values('importance',ascending=False).set_in
# importances6
```

DOWNSAMPLED

In [73]:

```
from sklearn.utils import resample

# setting up testing and training sets
X_train, X_test, y_train, y_test = train_test_split(x, y, test_size=0.25, rand

# concatenate our training data back together
X = pd.concat([x, y], axis=1)

# separate minority and majority classes
Non_health_Uptake= X[X['NHIF_health_insurance']==0]
health_Uptake= X[X['NHIF_health_insurance']==1]

# upsample minority
health_undersampled = resample(Non_health_Uptake,
                          replace=True, # sample with replacement
                          n_samples=len(health_Uptake), # match number in majo
                          random_state=2) # reproducible results

# combine majority and upsampled minority
undersampled = pd.concat([health _undersampled , health_Uptake])

# check new class counts
undersampled['NHIF_health_insurance'].value_counts()
```

Out[73]:

```
0    4126
1    4126
Name: NHIF_health_insurance, dtype: int64
```

In [74]:

```
N = undersampled['NHIF_health_insurance']
M= pd.get_dummies(undersampled.drop(['NHIF_health_insurance'], axis=1))
```

In [75]:

```
train_ratio = 0.7
validation_ratio = 0.15
test_ratio = 0.15
kfold = 5
x_train, x_test, y_train, y_test = train_test_split(M,N, test_size=0.15)
StratifiedKFold(n_splits=kfold)
# test is now 15% of the initial data set
# validation is now 15% of the initial data set
x_val, x_test, y_val, y_test = train_test_split(x_test, y_test, test_size=test_
#print(x_train, x_val, x_test
```

In [77]:
```
# Function to optimize model using gridsearch
def gridsearch(model, params,x_train, x_test, y_train, y_test, kfold):
    gs = GridSearchCV(model, params, scoring='accuracy', n_jobs=-1, cv=kfold)
    gs.fit(x_train, y_train)
    print ('Best params: ', gs.best_params_)
    print ('Best AUC on Train set: ', gs.best_score_)
    print( 'Best AUC on Test set: ', gs.score(x_test, y_test))

# Function to generate confusion matrix
def confmat(pred, y_test):
    conmat = np.array(confusion_matrix(y_test, pred, labels=[1,0]))
    conf = pd.DataFrame(conmat, index=['Yes', 'No'],
                         columns=['Predicted yes', 'Predicted no'])
    print( conf)

# Function to plot roc curve
def roc(prob, y_test):
    y_score = prob
    fpr = dict()
    tpr = dict()
    roc_auc=dict()
    fpr[1], tpr[1], _ = roc_curve(y_test, y_score)
    roc_auc[1] = auc(fpr[1], tpr[1])
    plt.figure(figsize=[7,7])
    plt.plot(fpr[1], tpr[1], label='Roc curve (area=%0.2f)' %roc_auc[1], linew
    plt.plot([1,0], [1,0], 'k--', linewidth=4)
    plt.xlim([0.0, 1.0])
    plt.ylim([0.0, 1.0])
    plt.xlabel('False Positive rate', fontsize=15)
    plt.ylabel('True Positive rate', fontsize=15)
    plt.title('ROC curve for Credit Default', fontsize=16)
    plt.legend(loc='Lower Right')
    plt.show()

def model(md, x_train, y_train,x_test, y_test):
    md.fit(x_train, y_train)
    pred = md.predict(x_test)
    #prob = md.predict_proba(x_test)[:,1]
    print( ' ' )
    print ('Accuracy on Train set: ', md.score(x_train, y_train))
    print( 'Accuracy on Test set: ', md.score(x_test, y_test))
    print( ' ')
    print(classification_report(y_test, pred))
    print( ' ')
    print('Confusion Matrix',confmat(pred, y_test))

    #roc(prob, y_test)
    return md
```

In [78]:
```
lg=LogisticRegression( penalty='l1',
    solver='liblinear',
    fit_intercept=True,
    random_state=2)
model_lg = model(lg, x_train, y_train,x_test, y_test)
```

```
Accuracy on Train set:  0.7707442258340462
Accuracy on Test set:  0.7350565428109854

              precision    recall  f1-score   support

           0       0.68      0.82      0.74       288
           1       0.81      0.66      0.73       331

    accuracy                           0.74       619
   macro avg       0.74      0.74      0.73       619
weighted avg       0.75      0.74      0.73       619


     Predicted yes  Predicted no
Yes            219           112
No              52           236
Confusion Matrix None
```

In [79]:
```
rf=RandomForestClassifier(random_state=2,n_estimators=300,min_samples_split=3,
model_rf = model(rf, x_train, y_train,x_test, y_test)
```

```
Accuracy on Train set:  0.9981465640148275
Accuracy on Test set:  0.7592891760904685

              precision    recall  f1-score   support

           0       0.71      0.82      0.76       288
           1       0.82      0.70      0.76       331

    accuracy                           0.76       619
   macro avg       0.76      0.76      0.76       619
weighted avg       0.77      0.76      0.76       619


     Predicted yes  Predicted no
Yes            233            98
No              51           237
Confusion Matrix None
```

In [80]:
```
DT=DecisionTreeClassifier(criterion='entropy', random_state=2)
model_DT = model(DT, x_train, y_train,x_test, y_test)
```

```
Accuracy on Train set:  1.0
Accuracy on Test set:  0.7334410339256866

              precision    recall  f1-score   support

           0       0.70      0.76      0.73       288
           1       0.77      0.71      0.74       331

    accuracy                           0.73       619
   macro avg       0.73      0.74      0.73       619
weighted avg       0.74      0.73      0.73       619


     Predicted yes  Predicted no
Yes            235            96
No              69           219
Confusion Matrix None
```

In [81]:
```
# feature selection with the best model from grid search
xgb = XGBClassifier(base_score=0.5, booster='gbtree', colsample_bylevel=1,
              colsample_bynode=1, colsample_bytree=1, gamma=0, gpu_id=-1,
              importance_type='gain', interaction_constraints='',
              learning_rate=0.2, max_delta_step=0, max_depth=6,
              min_child_weight=1, monotone_constraints='()',
              n_estimators=100, n_jobs=0, num_parallel_tree=1, random_state=2,
              reg_alpha=0, reg_lambda=1, scale_pos_weight=1, subsample=1,
              tree_method='exact', validate_parameters=1, verbosity=None)
model_xgb = model(xgb, x_train, y_train,x_test, y_test)
```

```
[08:11:16] WARNING: C:/Users/Administrator/workspace/xgboost-win64_release_1.
5.0/src/learner.cc:1115: Starting in XGBoost 1.3.0, the default evaluation me
tric used with the objective 'binary:logistic' was changed from 'error' to 'l
ogloss'. Explicitly set eval_metric if you'd like to restore the old behavio
r.

Accuracy on Train set:  0.8916452808668378
Accuracy on Test set:  0.7544426494345718

              precision    recall  f1-score   support

           0       0.71      0.80      0.75       288
           1       0.80      0.72      0.76       331

    accuracy                           0.75       619
   macro avg       0.76      0.76      0.75       619
weighted avg       0.76      0.75      0.75       619


     Predicted yes  Predicted no
Yes            237            94
No              58           230
Confusion Matrix None
```

In [82]:
```
clfs = {
'LogisticRegression' : lg,
'RandomForest': rf,
'XGBoost': xgb
}
#code block to test all models in clfs and generate a report
models_report = pd.DataFrame(columns = ['Model', 'Precision_score', 'Recall_sc

for clf, clf_name in zip(clfs.values(), clfs.keys()):
    clf.fit(x_train,y_train)
    y_pred = clf.predict(x_test)
    y_score = clf.score(x_test,y_test)

    #print('Calculating {}'.format(clf_name))
    t = pd.Series({
                     'Model': clf_name,
                     'Precision_score': metrics.precision_score(y_test, y_pred
                     'Recall_score': metrics.recall_score(y_test, y_pred,avera
                     'F1_score': metrics.f1_score(y_test, y_pred,average='macr
                     #'AUC':metrics.roc_auc_score(y_test, y_pred),
                     'Accuracy': metrics.accuracy_score(y_test, y_pred)}

                   )

    models_report = models_report.append(t, ignore_index = True)

models_report
```

```
[08:11:20] WARNING: C:/Users/Administrator/workspace/xgboost-win64_release_1.
5.0/src/learner.cc:1115: Starting in XGBoost 1.3.0, the default evaluation me
tric used with the objective 'binary:logistic' was changed from 'error' to 'l
ogloss'. Explicitly set eval_metric if you'd like to restore the old behavio
r.
```

Out[82]:

	Model	Precision_score	Recall_score	F1_score	Accuracy
**0**	LogisticRegression	0.743140	0.740538	0.734857	0.735057
**1**	RandomForest	0.763943	0.763422	0.759279	0.759289
**2**	XGBoost	0.756633	0.757312	0.754411	0.754443

In [83]:
```
probas_train = lg.predict_proba(x_train)
fpr_t, tpr_t, thresholds_t = roc_curve(y_train, probas_train[:, 1])
trai=auc(fpr_t, tpr_t)
# Testing Set
probas_ = lg.predict_proba(x_test)
fpr_lg, tpr_lg, thresholds = roc_curve(y_test, probas_[:, 1])
roc_auc_lg = auc(fpr_lg, tpr_lg)

train, = plt.plot(fpr_t, tpr_t, label = 'Train=' +str(round(trai,4)))
test, = plt.plot(fpr_lg, tpr_lg, label = 'Test=' +str(round(roc_auc_lg,4)))
plt.xlabel('False Positive Rate')
plt.ylabel('True Positive Rate')
plt.legend()
plt.show()

print('Area Under the Curve (AUC): ', roc_auc_lg)
```

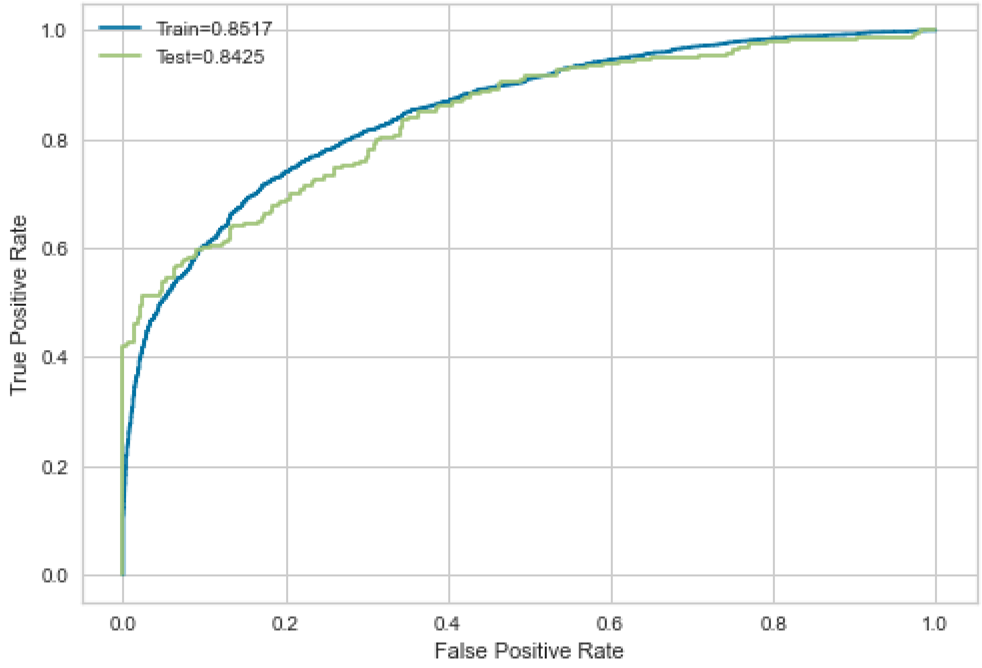


```
Area Under the Curve (AUC):  0.8424597180261834
```

In [84]:
```
probas_train = rf.predict_proba(x_train)
fpr_t, tpr_t, thresholds_t = roc_curve(y_train, probas_train[:, 1])
trai=auc(fpr_t, tpr_t)
# Testing Set
probas_ = rf.predict_proba(x_test)
fpr_rf, tpr_rf, thresholds = roc_curve(y_test, probas_[:, 1])
roc_auc_rf= auc(fpr_rf, tpr_rf)

train, = plt.plot(fpr_t, tpr_t, label = 'Train=' +str(round(trai,4)))
test, = plt.plot(fpr_rf, tpr_rf, label = 'Test=' +str(round(roc_auc_rf,4)))
plt.xlabel('False Positive Rate')
plt.ylabel('True Positive Rate')
plt.legend()
plt.show()

print('Area Under the Curve (AUC): ', roc_auc_rf)
```

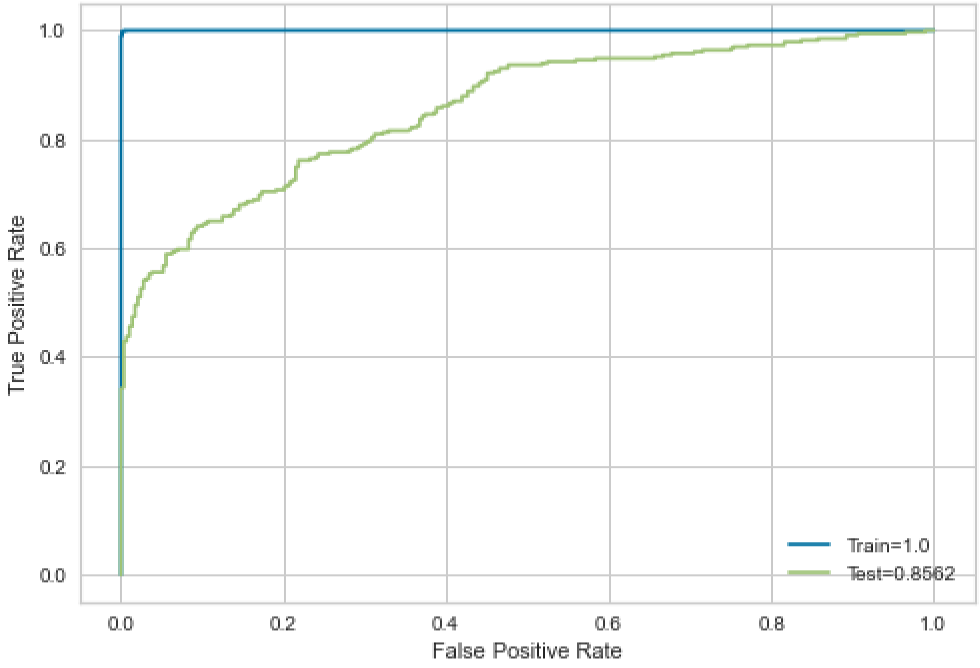


```
Area Under the Curve (AUC):  0.8562332158442431
```

In [85]:
```
probas_train = xgb.predict_proba(x_train)
fpr_t, tpr_t, thresholds_t = roc_curve(y_train, probas_train[:, 1])

# Testing Set
probas_ = xgb.predict_proba(x_test)
fpr_XGB, tpr_XGB, thresholds = roc_curve(y_test, probas_[:, 1])
roc_auc_XGB = auc(fpr_XGB, tpr_XGB)

train, = plt.plot(fpr_t, tpr_t, label = 'Train')
test, = plt.plot(fpr_XGB, tpr_XGB, label = 'Validation')
plt.xlabel('False Positive Rate')
plt.ylabel('True Positive Rate')
plt.legend()
plt.show()

print('Area Under the Curve for XGB: ', roc_auc_XGB)
```

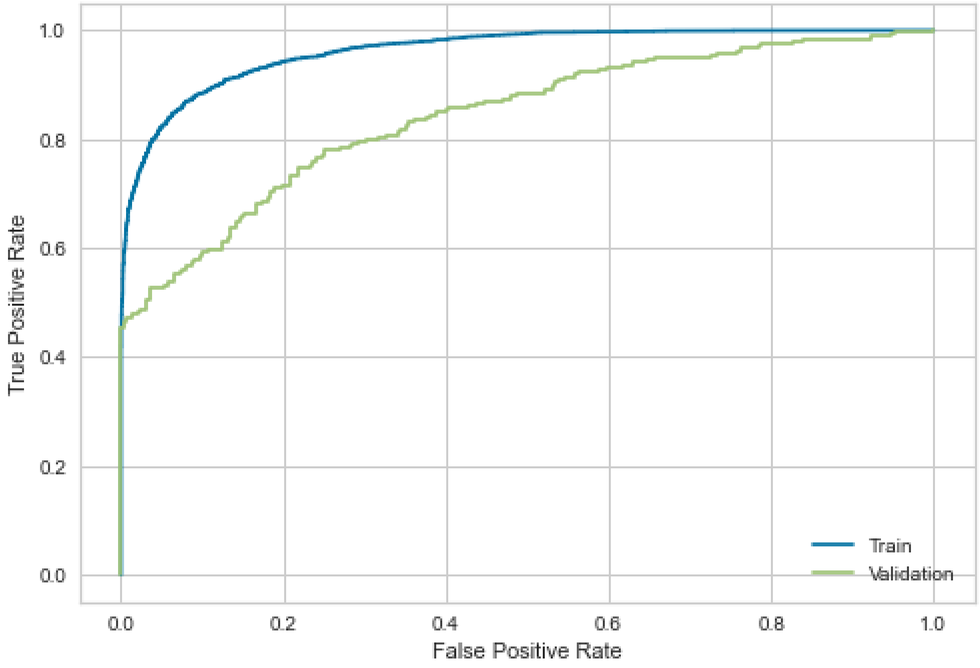


```
Area Under the Curve for XGB:  0.841515609264854
```

In [86]:
```
plt.figure(figsize=(7,7))
test, = plt.plot(fpr_lg, tpr_lg, label = 'logistic regression=' +str(round(roc_
test, = plt.plot(fpr_rf, tpr_rf, label ='Random forest=' +str(round(roc_auc_rf
test, = plt.plot(fpr_XGB, tpr_XGB, label = 'XGBoost=' +str(round(roc_auc_XGB,4
plt.plot([0,1],[0,1],'k--')
plt.xlabel('False Positive Rate')
plt.ylabel('True Positive Rate')
plt.title('Area Under Receiver Operating Characteristic Curve (AUC)',fontsize=
plt.annotate('At AUC of 0.5 the model hasa class \n separation capacity as good
                arrowprops=dict(facecolor='b', shrink=0.05),
                )
plt.legend(fontsize=17)
plt.grid()
plt.show()
```

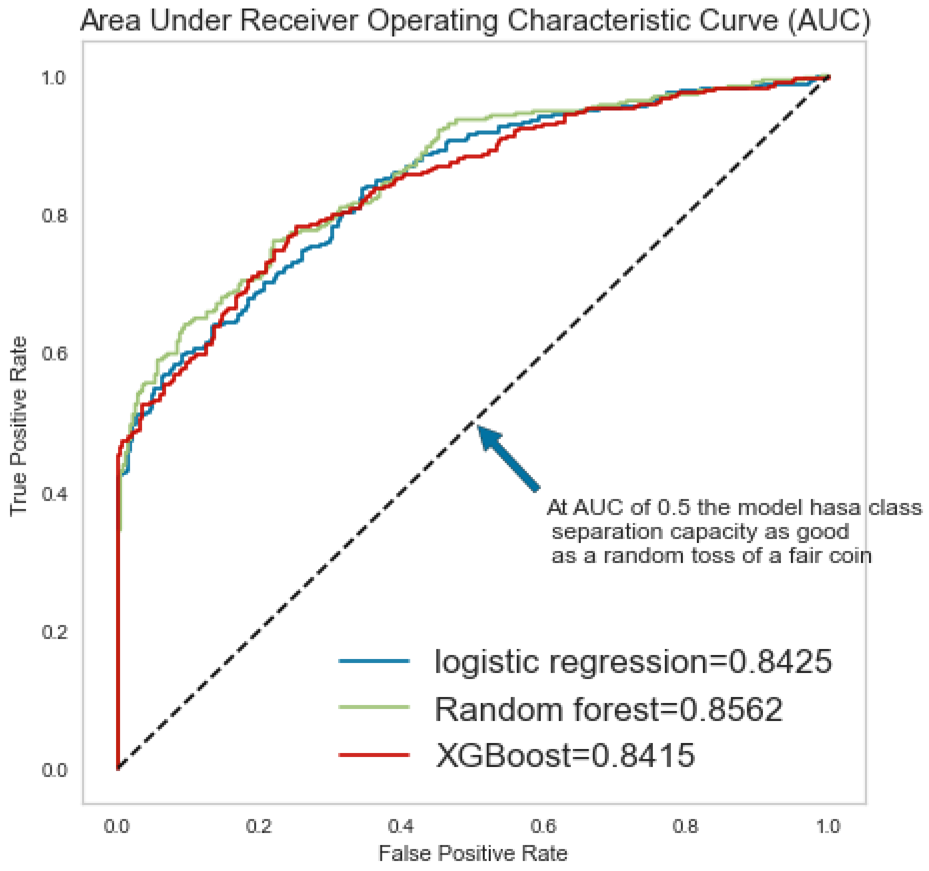

In [ ]:

In [ ]:

In [ ]:
